# Optical coherence tomography reveals retinal thinning in schizophrenia spectrum disorders

**DOI:** 10.1007/s00406-022-01455-z

**Published:** 2022-08-05

**Authors:** Emanuel Boudriot, Benedikt Schworm, Lenka Slapakova, Katharina Hanken, Iris Jäger, Marius Stephan, Vanessa Gabriel, Georgios Ioannou, Julian Melcher, Genc Hasanaj, Mattia Campana, Joanna Moussiopoulou, Lisa Löhrs, Alkomiet Hasan, Peter Falkai, Oliver Pogarell, Siegfried Priglinger, Daniel Keeser, Christoph Kern, Elias Wagner, Florian J. Raabe

**Affiliations:** 1 Department of Psychiatry and Psychotherapy, University Hospital, LMU Munich, Nußbaumstraße 7, 80336 Munich, Germany; 2 Department of Ophthalmology, University Hospital, LMU Munich, 80336 Munich, Germany; 3 International Max Planck Research School for Translational Psychiatry (IMPRS-TP), 80804 Munich, Germany; 4 Department of Psychiatry, Psychotherapy and Psychosomatics, Medical Faculty, University of Augsburg, 86156 Augsburg, Germany; 5 Max Planck Institute of Psychiatry, 80804 Munich, Germany; 6 NeuroImaging Core Unit Munich (NICUM), University Hospital, LMU Munich, 80336 Munich, Germany; 7 Munich Center for Neurosciences (MCN), LMU Munich, 82152 Planegg-Martinsried, Germany

**Keywords:** OCT, Angiography, Schizophrenia, Retina, Thickness, Perfusion

## Abstract

**Background:**

Schizophrenia spectrum disorders (SSDs) are presumed to be associated with retinal thinning. However, evidence is lacking as to whether these retinal alterations reflect a disease-specific process or are rather a consequence of comorbid diseases or concomitant microvascular impairment.

**Methods:**

The study included 126 eyes of 65 patients with SSDs and 143 eyes of 72 healthy controls. We examined macula and optic disc measures by optical coherence tomography (OCT) and OCT angiography (OCT-A). Additive mixed models were used to assess the impact of SSDs on retinal thickness and perfusion and to explore the association of retinal and clinical disease-related parameters by controlling for several ocular and systemic covariates (age, sex, spherical equivalent, intraocular pressure, body mass index, diabetes, hypertension, smoking status, and OCT signal strength).

**Results:**

OCT revealed significantly lower parafoveal macular, macular ganglion cell–inner plexiform layer (GCIPL), and macular retinal nerve fiber layer (RNFL) thickness and thinner mean and superior peripapillary RNFL in SSDs. In contrast, the applied OCT-A investigations, which included macular and peripapillary perfusion density, macular vessel density, and size of the foveal avascular zone, did not reveal any significant between-group differences. Finally, a longer duration of illness and higher chlorpromazine equivalent doses were associated with lower parafoveal macular and macular RNFL thickness.

**Conclusions:**

This study strengthens the evidence for disease-related retinal thinning in SSDs.

**Supplementary Information:**

The online version contains supplementary material available at 10.1007/s00406-022-01455-z.

## Introduction

Schizophrenia spectrum disorders (SSDs) are associated with significant global and widespread alterations in brain structure [1, 2], microstructure [3], and connectivity [4–6] and have a severe impact on cognition and social functioning [7]. Neurodevelopment is presumed to be atypical in SSDs [8], and several lines of evidence suggest that the regenerative capacity of the brain is impaired [7].

From an embryological perspective, the retina is part of the central nervous system (CNS). It does not just mimic many cellular processes of the healthy brain but also reflects various pathophysiological changes in neurodegenerative conditions, such as Alzheimer’s and Parkinson’s disease [9–11]. However, in contrast to the complex and deeply enmeshed neuronal networks of the brain, the highly structured cytoarchitecture of the human retina can be studied easily, quickly, and with very high resolution in vivo. Limitations in brain imaging encouraged researchers to harness the retina as a "window to the brain" [9] and use the non-invasive technology of optical coherence tomography (OCT) to explore retinal biomarkers of brain pathology [12].

In the last years, several pioneering studies have described alterations in retinal cytoarchitecture in SSDs [12–16]. A recent systematic review and meta-analysis, which included 23 studies with a total of 2079 eyes of patients with SSDs and 1571 eyes of healthy controls, revealed a reduction in peripapillary retinal nerve fiber layer (pRNFL) thickness, average macular thickness (MT), macular ganglion cell–inner plexiform layer (mGCIPL) thickness, and macular volume, as well as enlarged cup volume in SSDs [16]. However, the quality of previous studies was highly heterogeneous [17], and some reported negative results (e.g., [18, 19]). Findings across SSD studies were inconsistent as to whether the retinal nerve fiber layer (RNFL), macula, or other structures show abnormalities, and most past studies were too small to generate robust estimates of between-group differences [13].

Interestingly, the retina is also one of the few sites where the human microvasculature can be studied directly in vivo. Advanced OCT devices offer the possibility to visualize the capillary network by OCT angiography (OCT-A), which can reveal altered microvasculature in somatic diseases, such as diabetes or hypertension, even in the absence of retinopathy [20, 21]. There are only a few studies with small sample sizes of 12–28 patients with schizophrenia and 15–37 controls that have explored potential vascular changes in schizophrenia by OCT-A [22–25]. They indicated changes in several parameters within the patient groups, including reduced superficial vessel and perfusion density of the macula and larger foveal avascular zone (FAZ) area [25], decreased vessel density in the deep vascular plexus of the macula [23], and lower peripapillary vascular density in the temporal quadrant [22]. In one study, increased skeletonized vessel density in the superficial vascular plexus and increased vessel density and skeletonized vessel density in the choriocapillaris of the right eyes of patients with schizophrenia were detected [24].

Importantly, retinal investigations in mental illness face several limitations: It has been shown that age, sex, spherical equivalent, intraocular pressure (IOP), body mass index (BMI), diabetes, hypertension, smoking status [13, 21, 26–28], and OCT signal strength [29, 30] affect OCT and OCT-A measurements. Thus, effects of concomitant somatic conditions and cardiovascular risk factors, such as obesity, diabetes, hypertension, and smoking, that are over-represented in SSDs [13], and an altered microvascular state might have contributed to the reported retinal disturbances in SSDs.

In this study, we aimed to provide further evidence for the applicability of OCT and OCT-A as tools to study disease-related retinal processes in SSDs. Using an exploratory approach, we aimed to identify effects of SSDs on retinal structure and microvasculature by systematically controlling for potential covariates (age, sex, spherical equivalent, IOP, BMI, diabetes, hypertension, smoking status, and OCT signal strength) with a multivariate analysis strategy.

## Materials and methods

### Sample characteristics

This study was part of the Munich Clinical Deep Phenotyping study, an ongoing naturalistic study that started in October 2020 and focuses on schizophrenia. It was approved by the local ethics committee of the LMU Munich (approval number: 20-528) and registered in the German Clinical Trials Register (DRKS, registration ID: DRKS00024177). All participants provided written informed consent. This study provides a preliminary data analysis of participants that were enrolled between October 9, 2020, and July 21, 2021. Patients were recruited at the Department of Psychiatry and Psychotherapy, University Hospital, LMU Munich, Munich, Germany. Both in- and outpatients were considered for inclusion. Healthy controls were recruited from the local community via online advertisements, flyers, and personal referrals.

Inclusion criteria for patients were a diagnosis of schizophrenia, schizoaffective disorder, or brief psychotic disorder according to the Mini International Neuropsychiatric Interview (M.I.N.I.) [31], and for the healthy controls, no past or current psychiatric disorder according to the M.I.N.I. Exclusion criteria were a primary psychiatric disorder other than those mentioned above; age younger than 18 years or older than 65 years; a concurrent clinically relevant CNS disorder; a history of encephalitis, meningitis, or stroke; retinal pathology (pre-known or detected by OCT, for individual exclusion details see Supplemental Text); elevated IOP (≥ 21 mmHg); and pregnancy. Individual eyes were excluded at a spherical equivalent of less than or equal to -6 diopter (D) or greater than or equal to 6 D [32].

### Diagnosis and clinical assessment

All participants underwent the M.I.N.I. [31] for psychotic disorders studies, German version 7.0.2, according to *DSM-5* criteria. Symptom severity was assessed by the Positive and Negative Syndrome Scale (PANSS) [33]. Information on medications, disease history, concomitant conditions (e.g., diabetes, hypertension; defined as the presence of a medical diagnosis), height, weight, substance use in the past 7 days, and smoking status was collected through self-report and, if possible, verified by examining medical records. Current antipsychotic medication was converted to chlorpromazine equivalent doses (CPZeq) [34].

### OCT and OCT-A imaging

Eye examinations were performed at the Department of Ophthalmology, University Hospital, LMU Munich, Munich, Germany. Refraction and best corrected visual acuity (BCVA) were determined with an OCULUS/NIDEK AR 1-s autorefractor (OCULUS Optikgeräte GmbH, Wetzlar, Germany), and IOP, with a non-contact tonometer (OCULUS/NIDEK Tonoref II; OCULUS Optikgeräte GmbH, Wetzlar, Germany). For participants with previous refractive surgery, preoperative refraction was obtained from medical records. Before OCT imaging, most pupils were pharmacologically dilated with 0.5% tropicamide eye drops. Spectral-domain OCT and OCT-A scans of both eyes were then performed with a ZEISS CIRRUS HD-OCT 5000 with AngioPlex (Carl Zeiss Meditec AG, Jena, Germany), which has an axial resolution of 5 microns. The protocol comprised several scans: a 6 × 6 × 2 mm^3^ volume scan of the macula centered on the fovea, whereby each scan consisted of 128 brightness (B) scans with 512 amplitude (A) scans each; a 6 × 6 mm^2^ cube scan centered on the optic disc and consisting of 200 B-scans with 200 A-scans each; a 6 × 6 mm^2^ angiography scan centered on the fovea; and a 4.5 × 4.5 mm^2^ angiography scan of the peripapillary region. The angiography scans each consisted of 350 B-scan positions with 350 A-scans and two consecutive B-scans at each position. If necessary, individual scans were repeated to achieve adequate image quality. Scans were evaluated according to the OSCAR-IB criteria [32, 35] and excluded in case of notable artifacts. Only structural scans with a signal strength of at least 6 out of 10 and angiographies with a signal strength of at least 8 were accepted.

**Fig. 1:**
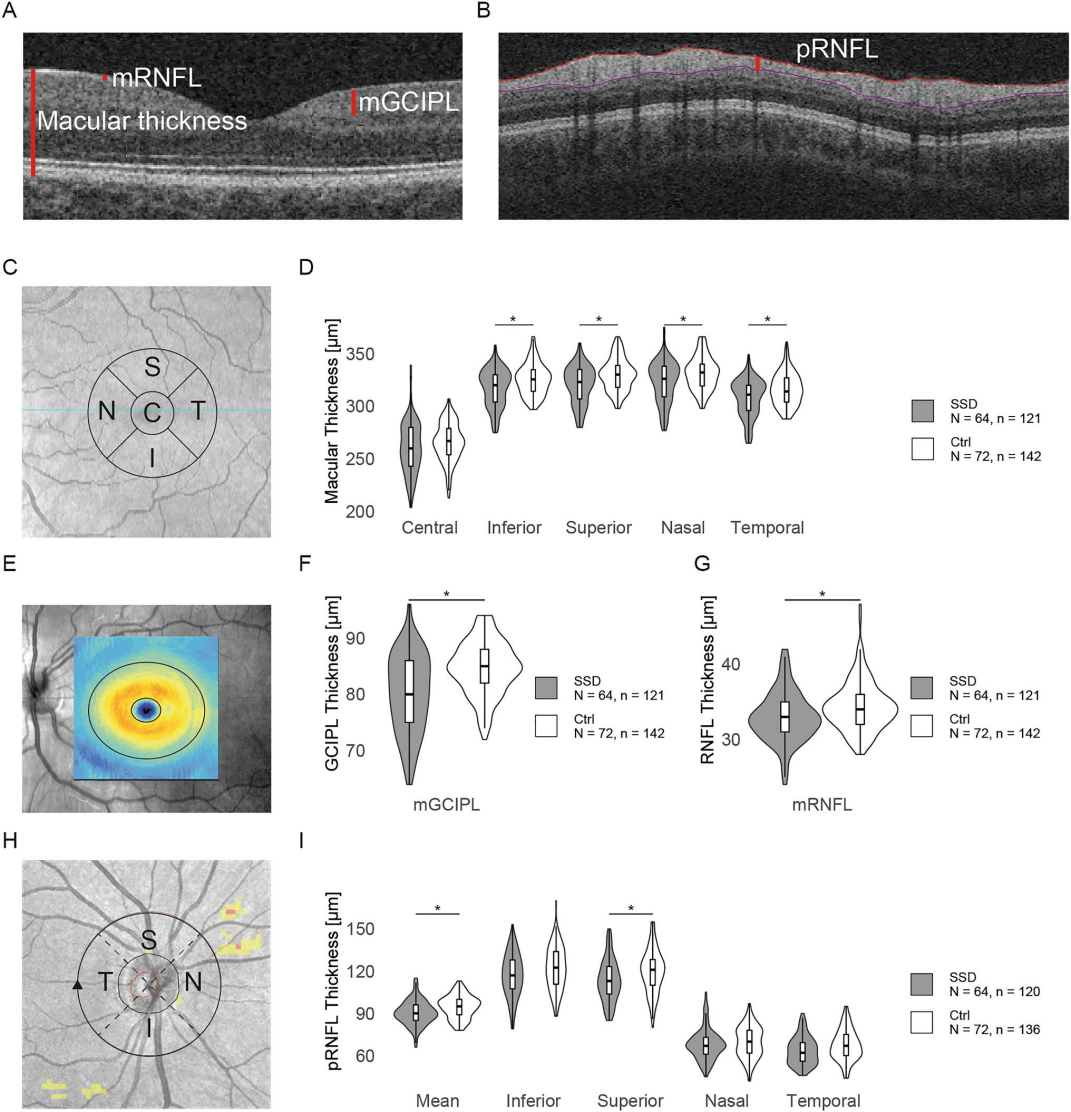
Optical coherence tomography reveals thinner retinal layers in patients with schizophrenia spectrum disorders compared with healthy controls.** A** Detail of a horizontal optical coherence tomography (OCT) brightness (B) scan of the macula. The red lines represent the macular thickness (MT), macular retinal nerve fiber layer (mRNFL), and combined ganglion cell–inner plexiform layer (mGCIPL). **B** Circular cut around the optic disc, illustrating the measurement of the peripapillary retinal nerve fiber layer (pRNFL; vertical red line). **C** OCT fundus image of the macular area of a left eye illustrating the central field (“C”) and the adjacent superior (“S”), temporal (“T”), inferior (“I”), and nasal (“N”) fields of the inner ring of the Early Treatment Diabetic Retinopathy Study (ETDRS) grid, where the macular thickness was measured. **D** Violin plots showing the distribution of the macular thickness within the central field (*p* = 0.43) and the inferior (*p* = 0.030), superior (*p* = 0.015), nasal (*p* = 0.016), and temporal (*p* = 0.041) fields of the inner ring of the ETDRS grid between the schizophrenia spectrum disorder (SSD) and the healthy control (Ctrl) group. **E** Fundus image of a left eye. The thicknesses of the mRNFL and mGCIPL were measured inside the area enclosed by the two concentric ellipses. **F** Distribution of the mGCIPL thickness in patients with SSDs and Ctrl (*p* = 0.008), illustrated with violin plots. **G** Distribution of the mRNFL thickness in patients with SSDs and Ctrl (*p* = 0.008), illustrated with violin plots. **H** Illustration of the pRNFL measurement circle (black) for a right eye. Values were obtained for the mean and the temporal (“T”), superior (“S”), nasal (“N”) and inferior (“I”) quadrants. **I** Violin plots comparing the distribution of the mean pRNFL thickness in patients with SSDs and Ctrl (*p* = 0.021) and pRNFL thickness in the inferior (*p* = 0.54), superior (*p* = 0.018), nasal (*p* = 0.31), and temporal (*p* = 0.42) quadrants. If available, the measurements of both eyes are each included as separate observations. *p* values were obtained with additive mixed models and are false discovery rate adjusted. *N*, number of participants; *n*, number of eyes; *p < 0.05. *GCIPL* ganglion cell–inner plexiform layer; *mGCIPL* macular GCIPL; *RNFL* retinal nerve fiber layer *mRNFL* macular RNFL; *pRNFL* peripapillary RNFL

OCT data were automatically analyzed by the instrument's software (version 11.0.0.29946), and several parameters were evaluated in detail. The software calculated the MT—which equates to the distance between the internal limiting membrane and the posterior part of the retinal pigment epithelium (Fig. 1A)—according to the Early Treatment Diabetic Retinopathy Study (ETDRS) grid. Here, the retina is divided into 9 sectors that form inner and outer rings with outer diameters of 3 and 6 mm, respectively, around a 1-mm-diameter central foveal field. For the present work, we used only the values for the central and four adjacent subfields within the inner parafoveal ring (Fig. 1C). Mean mGCIPL and macular RNFL (mRNFL) thicknesses were determined by the instrument’s software within an elliptical ring around the fovea with an inner diameter of 1 mm vertically and 1.2 mm horizontally and outer diameters of 4 mm and 4.8 mm (Fig. 1A, E). To determine pRNFL thickness (Fig. 1B), a circle with a radius of 1.73 mm was placed around the optic disc. We assessed the mean value for the whole pRNFL and the separate values for the inferior, superior, nasal, and temporal quadrants (Fig. 1H). Automatic layer segmentation was checked for all scans. Scans with segmentation errors were excluded.

**Fig. 2:**
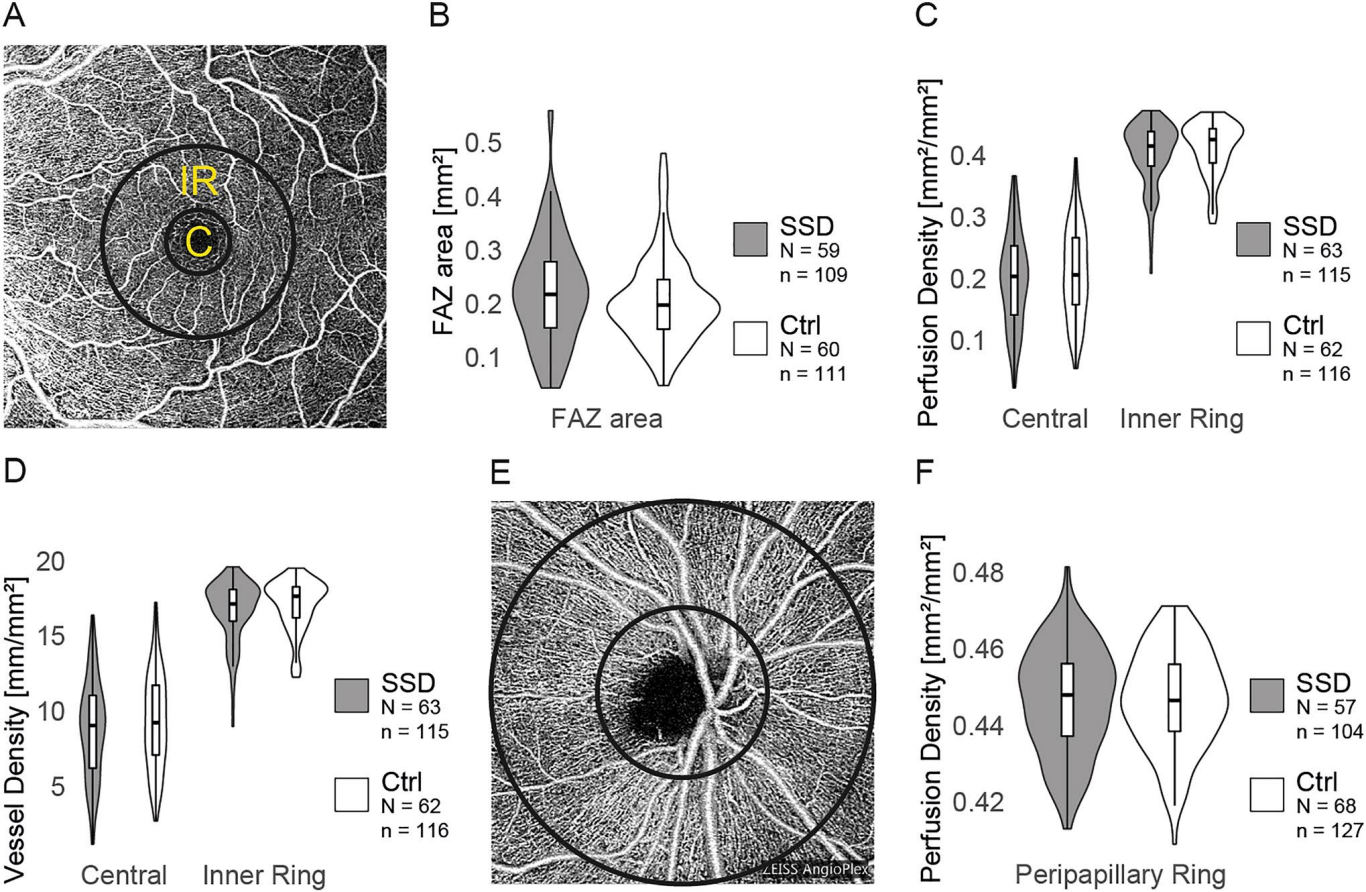
Illustration of the coherence tomography angiography investigations and comparison between patients with schizophrenia spectrum disorders and healthy controls. **A** Exemplary en face image of the superficial vascular plexus of the left macula. The central field (“C”) contains the foveal avascular zone (FAZ) and is surrounded by the inner ring (“IR”) of the Early Treatment Diabetic Retinopathy Study (ETDRS) grid. **B** Violin plots comparing the distribution of the FAZ size in patients with schizophrenia spectrum disorders (SSDs) and healthy controls (Ctrl; *p* = 0.43). **C** Violin plots comparing the distribution of the perfusion density in the macular area in patients with SSDs and Ctrl, separately for the central field (*p* = 0.96) and the inner ring (*p* = 0.66). **D** Distribution of the vessel density in the central field (*p* = 0.96) and the inner ring (*p* = 0.55) of the ETDRS grid, illustrated with violin plots. **E** En face image of a papillary optical coherence tomography angiography scan. The two black circles illustrate the annulus in which the peripapillary perfusion density was measured. **F** Violin plots comparing the distribution of the peripapillary perfusion density between patients with SSDs and Ctrl (*p* = 0.54). If available, the measurements of both eyes are each included as separate observations. *p* values were obtained with additive mixed models and are false discovery rate adjusted. *N*, number of participants; *n*, number of eyes; *p < 0.05. *FAZ* foveal avascular zone

OCT-A macular perfusion parameters included the perfusion and vessel densities of the superficial vascular plexus (Fig. 2A). Perfusion density was defined as the proportion of the area with blood flow, and vessel density, as the total length of all blood vessels per area. The software analyzed the corresponding values within the central foveal subfield, which included the FAZ, and inside the surrounding inner ring of the ETDRS grid. For peripapillary angiographies, perfusion density in the radial peripapillary capillary network was measured within an annulus with an outer diameter of 4.5 mm around the optic disc (Fig. 2E). Furthermore, the FAZ size was determined automatically by the software. The automatic FAZ detection was checked in each case and manually corrected on the OCT device if necessary. Individual scans were excluded from the FAZ analysis if the FAZ was not reasonably delineable, e.g., in anatomical variations in which the inner nuclear layer was not completely absent [36].

**Fig. 3:**
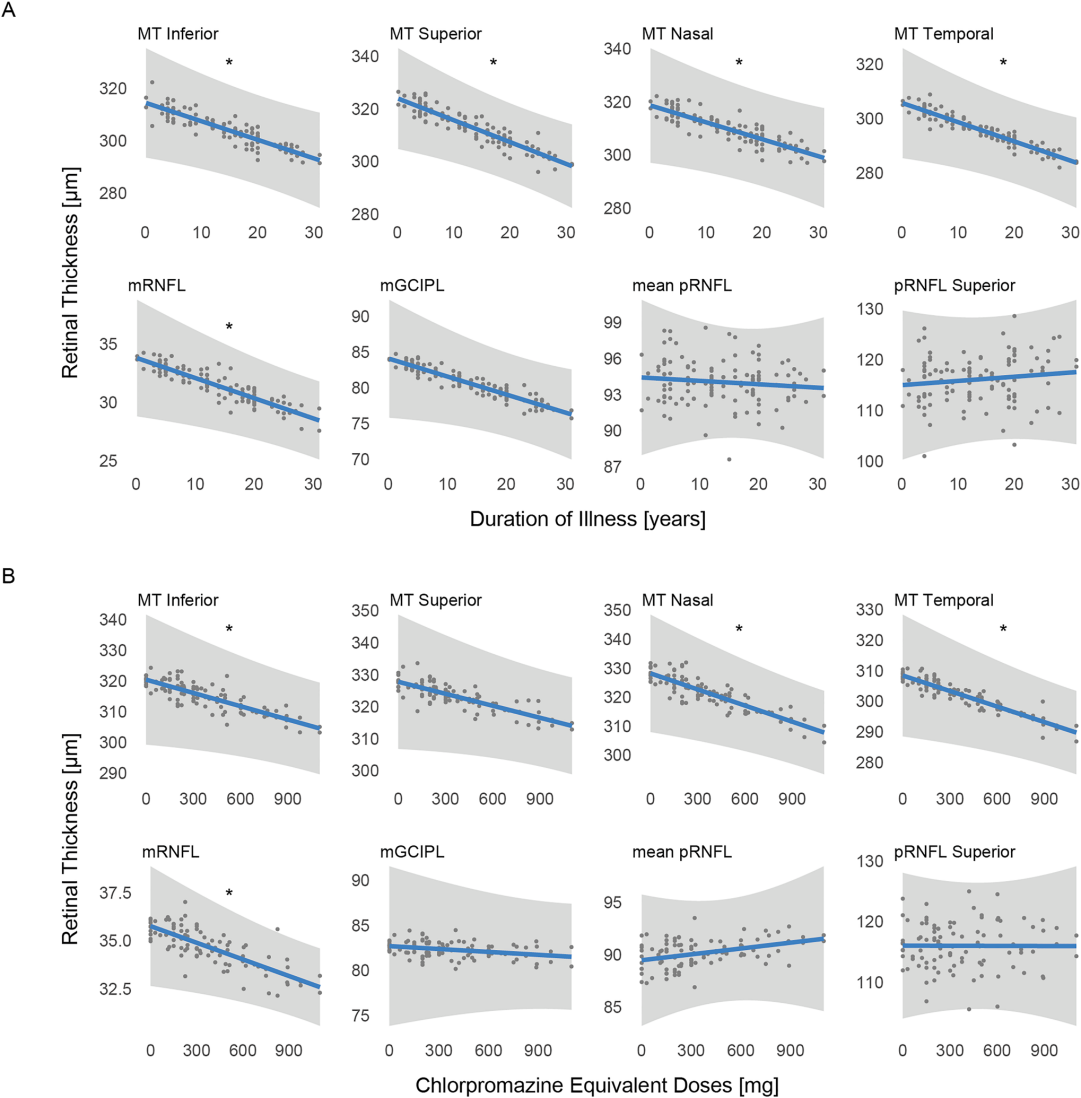
Association between retinal measures and clinical disease features. **A** Association of duration of illness with significantly altered optical coherence tomography (OCT) parameters, estimated with additive mixed models. The plots show how the expected values of the outcome variables (blue lines) change as a function of the duration of illness when all other model terms are held fixed. Included are grey 95% confidence bands and dots for the partial residuals. *p < 0.05. **B** Association of chlorpromazine equivalent doses with significantly altered OCT parameters, estimated with additive mixed models. The plots show how the expected values of the outcome variables (blue lines) change as a function of the chlorpromazine equivalent doses when all other model terms are held fixed. Included are grey 95% confidence bands and dots for the partial residuals. *p < 0.05. *MT Central* macular thickness in the central subfield; *MT Inferior* macular thickness in the inner inferior subfield; *MT Superior* macular thickness in the inner superior subfield; *MT Nasal* macular thickness in the inner nasal subfield; *MT Temporal* macular thickness in the inner temporal subfield; *mGCIPL* macular ganglion cell–inner plexiform layer; *mRNFL* macular retinal nerve fiber layer; *pRNFL* peripapillary retinal nerve fiber layer

### Statistical analysis

Statistical analyses were performed with R, version 4.1.1 [37]. Group differences in sample characteristics were explored with Fisher's exact test for categorical variables and with Welch's *t* test for normally distributed and Mann–Whitney *U* test for non-normally distributed continuous variables [38, 39]. Normality within groups was assessed with the Shapiro–Wilk test.

We studied the association between SSDs and retinal parameters from OCT and OCT-A measures with additive mixed models (AMMs). These models enable the inclusion of non-linear smooth effects of multiple covariates [40]. The regression models were estimated with the *gam* function of the *mgcv* package [41]. Both eyes (i.e., oculus uterque, OU) were included if available. We adjusted for the correlation of the measurements of each participant’s eyes by including a random intercept for participant identification number. It has been reported that age, sex, spherical equivalent, IOP, BMI, diabetes, hypertension, smoking status [13, 21, 26–28], and OCT signal strength [29, 30] affect OCT and OCT-A measurements. These variables were, therefore, considered as covariates. Non-linear effects were estimated on a P-spline basis with 10 basis functions; residuals were visually checked and showed no substantial deviation from the model assumptions. To address possible inter-eye differences, we additionally fitted separate additive models for the right (i.e., oculus dexter, OD) and left (i.e., oculus sinister, OS) eyes and included the mentioned covariates. The resulting *p* values of the group effects of the OU, OD, and OS models were jointly adjusted for multiple testing within one Benjamini–Hochberg procedure [42].

Next, we performed exploratory post hoc analyses and used separate additive mixed models to address the association of duration of illness, CPZeq, and lifetime history of treatment with clozapine with those parameters that were significantly altered in the OU analysis, controlling for the aforementioned covariates. Duration of illness and CPZeq were included as linear predictors. Because of the primarily hypothesis-generating nature of these post hoc analyses, they were not corrected for multiple testing.

For all analyses, a *p* value of less than or equal to 0.05 was considered statistically significant.

## Results

### Demographic and clinical characteristics

83 patients with SSDs and 89 healthy controls underwent OCT. After excluding ineligible scans (see Supplemental Text), the following scans from a total of 126 eyes of 65 patients and 143 eyes of 72 controls were available: macular OCT scans of 121 eyes of 64 patients and 142 eyes of 72 controls, papillary OCT scans of 120 eyes of 64 patients and 136 eyes of 72 controls, macular OCT-A scans of 115 eyes of 63 patients and 116 eyes of 62 controls, and papillary OCT-A scans of 104 eyes of 57 patients and 127 eyes of 68 controls. Sex distribution was not significantly different between groups (Table 1). Mean age was 4.64 years higher in patients, and mean BMI was 6.88 kg/m^2^ higher. Nearly half (48%) of patients were smokers, compared with only 15% of controls. Five patients and none of the controls had a concomitant diagnosis of type 2 diabetes. The groups did not differ significantly in the frequency of hypertension. We observed that within our study cohort the mean spherical equivalent was 0.67 D lower in patients than in controls, and the mean IOP was 0.64 mmHg higher. BCVA showed no significant differences. OCT signal strength in the optic disc scans was slightly higher in patients than in controls, but no significant differences were observed for the other scans (Table 1).

**Table 1:** Cohort characteristics

Sociodemographic variables	SSD group		Ctrl group		
	Mean ± SD	*n*	Mean ± SD	*n*	*p*
Age, years	39.29 ± 10.8	65	34.65 ± 11.35	72	0.007^*b*^
	*n* (%)		*n* (%)		
Sex, male:female	42:23 (65%)		36:36 (50%)		0.12^*a*^
	Mean ± SD	*n*	Mean ± SD	*n*	
BMI, kg/m^2^	30.41 ± 6.83	65	23.53 ± 3.03	72	< 0.001^*b*^
	*n* (%)		*n* (%)		
Diabetes^*c*^, yes:no	5:60 (8%)		0:72 (0%)		0.022^*a*^
Hypertension^*c*^, yes:no	9:56 (14%)		5:67 (7%)		0.26^*a*^
Smoking status, yes:no	31:34 (48%)		11:61 (15%)		< 0.001^*a*^

Treatment and severity of illness	Mean ± SD	*n*	Mean ± SD	*n*	*p*
CPZeq, mg	366.03 ± 273.46	55	–	–	–
Duration of illness, years	13.69 ± 7.81	63	–	–	–
PANSS positive symptoms	11.46 ± 4.15	65	7.14 ± 0.42	72	< 0.001^*b*^
PANSS negative symptoms	11.57 ± 4.68	65	7.53 ± 1.09	72	< 0.001^*b*^
PANSS general symptoms	24.86 ± 7.32	65	16.89 ± 1.42	72	< 0.001^*b*^
PANSS total score	47.75 ± 14.47	65	31.56 ± 2.26	72	< 0.001^*b*^
	*n* (%)		–		–
Lifetime clozapine treatment, yes:no	30:34 (47%)		–		–

Ophthalmic variables	Mean ± SD	*n* (eyes)	Mean ± SD	*n* (eyes)	*p*
Spherical equivalent, D	−1.64 ± 1.64	126	−0.97 ± 1.60	143	< 0.001^*b*^
IOP, mmHg	13.59 ± 2.72	126	12.95 ± 2.75	143	0.028^*b*^
BCVA	1.18 ± 0.15	126	1.20 ± 0.13	143	0.36^*b*^
Signal strength OCT, macula	8.59 ± 0.95	121	8.42 ± 0.99	142	0.15^*b*^
Signal strength OCT, optic disc	7.83 ± 0.91	120	7.57 ± 0.81	136	0.020^*b*^
Signal strength OCT-A, macula	8.90 ± 0.77	115	8.91 ± 0.79	116	0.88^*b*^
Signal strength OCT-A, optic disc	9.13 ± 0.80	104	8.97 ± 0.80	127	0.12^*b*^

Diagnosis (*DSM-5*)	*n* (%)		–		–
Schizophrenia	47 (72%)		–		–
Schizoaffective disorder	17 (26%)		–		–
Brief psychotic disorder	1 (2%)		–		–

*BCVA* best corrected visual acuity; *BMI* body mass index; *CPZeq* chlorpromazine equivalent doses; *Ctrl* healthy controls; *D* diopters; *IOP* intraocular pressure; *n* number of observations; *OCT* optical coherence tomography; *OCT-A* optical coherence tomography angiography; *p p* value; *PANSS* Positive and Negative Syndrome Scale; *SD* standard deviation; *SSD* schizophrenia spectrum disorder

^*a*^Fisher’s exact test

^*b*^Mann–Whitney *U* test

^*c*^Information regarding concomitant diagnoses of somatic conditions was collected through self-report and by examining medical records

Among the patients, mean duration of illness was 13.69 years (*SD* = 7.81) and mean CPZeq was 366.03 mg (*SD* = 273.46). Ten patients had missing data for CPZeq, and two, for duration of illness. Most patients were clinically stable according to the PANSS; mean PANSS total score was 47.75 (*SD* = 14.47). According to the M.I.N.I., most patients had a diagnosis of schizophrenia (72%), followed by schizoaffective disorder (26%). Only one patient, a 63-year-old woman with first-episode psychosis, was diagnosed with brief psychotic disorder (Table 1). No healthy control and only one patient reported use of cannabis within the 7 days before the examination.

### OCT reveals retinal thinning in SSDs

We examined the retinal cytoarchitecture in both groups by OCT (Fig. 1). To estimate the impact of SSDs on the measurements, we fitted additive mixed models, which enable adjustment for non-linear predictor variables [40]. We included age, sex, spherical equivalent, IOP, BMI, diabetes, hypertension, smoking status, and OCT signal strength as covariates in all analyses; except for diabetes and smoking status, all covariates were significantly associated with at least some of the OCT outcome measures. More detailed information of the partial effects of the included covariates on the respective OCT measurements is provided in the Supplemental Model Reports. The partial effects of the covariates on pRNFL thickness are highlighted as an example (Fig. S1).

Table 2 reports and Fig. S2A illustrates the estimates of the group effect on OCT measurements. Despite accounting for the effects of the aforementioned covariates, the analysis revealed a robust and significant thinning of the parafoveal MT in SSD (Fig. 1D; Table 2). MT was lower in the SSD group in the inferior (estimate [95% CI] = −8.80 µm [−15.62, −1.98]; *p* = 0.030), superior (estimate [95% CI] = −11.13 µm [−18.48, −3.78]; *p* = 0.015), nasal (estimate [95% CI] = −10.21 µm [−17.25, −3.18]; *p* = 0.016), and temporal (estimate [95% CI] = −8.74 µm [−15.89, −1.59]; *p* = 0.041) fields of the inner ring of the ETDRS grid, but no significant differences between groups were found in the central foveal field (*p* = 0.43). Within the macular area, we found thinner mRNFL (estimate [95% CI] = −2.40 µm [−3.78, −1.03]; *p* = 0.008) and mGCIPL (estimate [95% CI] = −4.46 µm [−6.95, −1.97]; *p* = 0.008; Fig. 1F, G) in SSD. Mean pRNFL thickness (Fig. 1I) was also lower in patients (estimate [95% CI] = −4.72 µm [−8.14, −1.30]; *p* = 0.021), driven mainly by a strong effect in the superior quadrant (estimate [95% CI] = −8.54 µm [−14.54, −2.55]; *p* = 0.018), whereas SSD had no significant effect on pRNFL thickness in the inferior (*p* = 0.54), nasal (*p* = 0.31), or temporal (*p* = 0.42) quadrants.

**Table 2:** Descriptive statistics and estimates for the retinal measures (oculus uterque)

	SSD		Ctrl		Estimate [95% CI]	*n*	*p*	*p* (FDR adj.)	
	Mean	SD	Mean	SD					
*OCT measurements*									
MT, central subfield (µm)	260.27	23.71	265.75	18.11	−4.3611 [−12.2245, 3.5023]	263	0.2792	0.4307	*ns*
MT, inner inferior subfield (µm)	317.33	17.62	325.47	14.79	−8.8016 [−15.6197, −1.9835]	263	0.0127	0.0297	*
MT, inner superior subfield (µm)	320.30	17.81	329.94	15.07	−11.1310 [−18.4817, −3.7803]	263	0.0036	0.0149	*
MT, inner nasal subfield (µm)	323.04	19.25	331.35	15.44	−10.2115 [−17.2475, −3.1754]	263	0.0052	0.0165	*
MT, inner temporal subfield (µm)	307.82	17.49	316.07	15.27	−8.7382 [−15.8902, −1.5862]	263	0.0181	0.0408	*
mRNFL thickness (µm)	32.98	3.34	34.39	3.29	−2.4031 [−3.7762, −1.0300]	263	0.0008	0.0079	*
mGCIPL thickness (µm)	80.17	6.90	84.66	4.75	−4.4632 [−6.9520, −1.9743]	263	0.0006	0.0079	*
pRNFL thickness, mean (µm)	90.44	8.66	94.71	8.08	−4.7171 [−8.1356, −1.2987]	256	0.0078	0.0211	*
pRNFL thickness, inferior (µm)	117.12	15.28	122.06	15.24	−2.4275 [−8.2082, 3.3533]	256	0.4120	0.5412	*ns*
pRNFL thickness, superior (µm)	113.33	14.85	119.86	15.35	−8.5430 [−14.5390, −2.5471]	256	0.0060	0.0180	*
pRNFL thickness, nasal (µm)	67.84	10.81	69.59	11.14	−3.6164 [−8.6257, 1.3928]	256	0.1595	0.3076	*ns*
pRNFL thickness, temporal (µm)	63.46	9.86	67.43	10.92	−2.6159 [−7.0151, 1.7833]	256	0.2460	0.4151	*ns*
*OCT-A measurements*									
FAZ area (mm^2^)	0.22	0.10	0.21	0.08	0.0289 [−0.0227, 0.0806]	220	0.2743	0.4307	*ns*
Perfusion density, central (mm^2^/mm^2^)	0.20	0.08	0.21	0.07	0.0014 [−0.0258, 0.0285]	231	0.9211	0.9565	*ns*
Perfusion density, inner ring (mm^2^/mm^2^)	0.40	0.05	0.41	0.04	−0.0047 [−0.0202, 0.0107]	231	0.5487	0.6584	*ns*
Vessel density, central (mm/mm^2^)	8.82	3.22	9.33	3.18	0.0671 [−1.0992, 1.2333]	231	0.9104	0.9565	*ns*
Vessel density, inner ring (mm/mm^2^)	16.73	2.01	17.07	1.71	−0.2402 [−0.8438, 0.3634]	231	0.4364	0.5481	*ns*
Perfusion density, peripapillary (mm^2^/mm^2^)	0.45	0.01	0.45	0.01	0.0023 [−0.0033, 0.0079]	231	0.4210	0.5412	*ns*

*Ctrl* healthy controls; *FAZ* foveal avascular zone; *mGCIPL* macular ganglion cell–inner plexiform layer; *mRNFL* macular retinal nerve fiber layer; *MT* macular thickness; *n* number of eyes (SSDs and Ctrl); *ns* not significant; *p*, *p* value; *p* (FDR adj.), false discovery rate adjusted *p* value; *pRNFL* peripapillary retinal nerve fiber layer; *SD* standard deviation; *SSD* schizophrenia spectrum disorder. *p < 0.05

Transferring the estimated effects to an average male, nonsmoking patient without diabetes or hypertension, with all other covariates set to the SSD group median, the following percentage changes would be expected compared with a psychiatrically healthy control with otherwise similar characteristics: −2.7% for the inferior, −3.3% for the superior, −3.0% for the nasal, and −2.7% for the temporal inner MT; −7.0% for the mRNFL; −5.3% for the mGCIPL; and −5.0% for the mean and −7.0% for the superior pRNFL thickness.

Next, we fitted separate models for the right (OD) and left (OS) eyes. These analyses yielded very similar results compared to the OU analysis and the same parameters were significantly altered (Fig. S2; Table S1).

In summary, regardless of the effects of the various ocular and systemic covariates on retinal thickness, we observed widespread retinal thinning in SSDs.

### Investigating the retinal microvasculature with OCT-A

To assess whether the observed retinal thinning in SSDs could be partly explained by an altered vascular state, OCT-A data were analyzed with additive mixed models (Fig. 2; Table 2) and, in line with the OCT analysis, age, sex, spherical equivalent, IOP, BMI, diabetes, hypertension, smoking status, and signal strength were included as covariates in all analyses. No differences between groups were found for perfusion density in the central foveal field (*p* = 0.96), in the 3-mm-diameter parafoveal ring (*p* = 0.66; Fig. 2C), or in the peripapillary area (*p* = 0.54; Fig. 2F) or for central (*p* = 0.96) or parafoveal (*p* = 0.55) vessel density (Fig. 2D). Moreover, the size of the FAZ did not differ between groups (*p* = 0.43; Fig. 2B). Table 2 reports and Fig. S2B illustrates the estimates and confidence intervals for these non-significant effects of SSD.

In addition, the vascular parameters were found to be associated (to varying degrees) with BMI, smoking status, sex, and OCT-A signal strength (Supplemental Model Reports). Similar to OCT data, the right and left eye exhibited comparable states (Fig. S2; Table S1).

### Association of retinal thickness with clinical disease-related parameters

Last, we performed exploratory post hoc analyses to assess whether the retinal measures that were significantly altered in the SSD group in this study (only OCT measures) were associated with clinically relevant parameters by controlling for age, sex, spherical equivalent, IOP, BMI, diabetes, hypertension, smoking status, and signal strength as covariates.

Table S2 reports the estimates for the effects of duration of illness and CPZeq. Interestingly, although we controlled for covariate effects (including age), longer duration of illness (in years) was significantly associated with thinner MT in the inferior (estimate [95% CI] = −0.7078 µm/year [−1.2977, −0.1180]; *p* = 0.022), superior (estimate [95% CI] = −0.8307 µm/year [−1.4156, −0.2458]; *p* = 0.007), nasal (estimate [95% CI] = −0.6398 µm/year [−1.2503, −0.0292]; *p* = 0.045), and temporal (estimate [95% CI] = −0.7085 µm/year [−1.3297, −0.0874]; *p* = 0.030) parafoveal fields and with thinner mean mRNFL thickness (estimate [95% CI] = −0.1729 µm/year [−0.3025, −0.0432]; *p* = 0.012; Fig. 3). Moreover, higher CPZeq (in mg) was significantly associated with lower inferior (estimate [95% CI] = −0.0144 µm/mg [−0.0282, −0.0006]; *p* = 0.047), nasal (estimate [95% CI] = −0.0186 µm/mg [−0.0318, −0.0053]; *p* = 0.009), and temporal (estimate [95% CI] = −0.0169 µm/mg [−0.0302, −0.0037]; *p* = 0.016) parafoveal MT and mRNFL thickness (estimate [95% CI] = −0.0029 µm/mg [−0.0052, −0.0005]; *p* = 0.020; Fig. 3). Neither duration of illness nor CPZeq was significantly associated with mGCIPL or pRNFL thickness.

As a proxy for treatment resistance, we further assessed the effect of lifetime history of treatment with clozapine on OCT measures. The additive mixed models revealed a significant negative association with mean pRNFL thickness (estimate [95% CI] = −4.64 µm [−8.12, −1.15]; *p* = 0.012; Fig. S3; Table S3).

## Discussion

This study presents a preliminary exploratory analysis of data from the ongoing Munich Clinical Deep Phenotyping Study. We systematically investigated the retina in a large cohort of patients with SSDs and healthy controls with the aims to explore differences in retinal thickness with OCT and to evaluate the retinal microvascular state with OCT-A by controlling for covariates associated with retinal alterations (age, sex, spherical equivalent, IOP, BMI, diabetes, hypertension, smoking status, and OCT signal strength). The multivariate analyses presented here revealed that SSDs were significantly associated with lower parafoveal macular, mGCIPL, mRNFL, and pRNFL thickness. In contrast, we could not detect accompanying microvascular alterations in SSDs regarding macular or peripapillary perfusion density, macular vessel density, and size of the foveal avascular zone.

The lack of between-group differences in the OCT-A parameters contrasts with previous smaller studies that showed several alterations in OCT-A parameters in SSDs [18, 22–24]. Of note, the previous findings were quite heterogeneous and ranged from reduced [18, 22, 23] to increased [24] perfusion in SSDs. Hence some previous OCT-A studies reported contradictory findings as to whether retinal alterations affect both eyes [25] or only one eye [24], we performed separate analyses for the right and left eyes that showed no relevant differences between the two eyes in either retinal thinning or the microvascular state.

Of note, several OCT-A studies in SSDs used different devices and image processing methods and are, therefore, not directly comparable with our study. A recent study of 28 patients with SSDs and 37 healthy controls that used the same OCT device as we did found that patients had lower macular perfusion density and larger FAZ areas in both eyes, as well as lower left macular vessel density [25]. However, the study used a different scanning protocol that had a higher resolution than ours, because it covered a smaller area of 3 × 3 mm^2^ and each A-scan and B-scan was separated by 12.2 microns, whereas the A-scans and B-scans in our 6 × 6 mm^2^ protocol were separated by 17.1 microns; in addition, the protocol had more B-scan repetitions per position (4 vs 2) [43, 44]. Therefore, the measurements obtained with our 6 × 6 mm^2^ scan might have lower repeatability [44]. OCT-A is a novel technology and still prone to artifacts [45–47]. This might explain part of the heterogeneity of previous findings. In the future, more sensitive OCT-A devices might be able to detect more subtle changes. A further technical limitation of our OCT-A analysis is that we included only the superficial vascular plexus. A previous smaller study that investigated the deeper vascular layers observed some increased vessel density and skeletonized vessel density in the choriocapillaris in SSDs [24]. Of interest is that other recent OCT studies found no differences in choroidal thickness between patients with SSDs and healthy controls [48–50]. A previous OCT-A study in SSDs found the most prominent differences for patients with early disease [24], whereas another study found no differences between first-episode and multi-episode patients [25]. Importantly, the patients in our study, which could not reveal any SSD-driven alterations of the retinal microvasculature, were mostly chronically ill.

Despite some technical limitations of our OCT-A investigation, we included only high-quality scans in our study and were able to draw on a large and well-powered data set. Our negative results challenge the positive findings of previous studies that investigated retinal microvasculature in SSDs by OCT-A.

Effects of systemic diseases, smoking, or obesity [13] and neuroinflammatory processes [25] have been postulated as possible etiologies of retinal alterations in SSDs. Importantly, our finding of retinal thinning was robust even after controlling for various covariates including cardiovascular risk factors and, moreover, was not associated with altered retinal microvasculature. Thus, we presume that the retinal thinning observed in our SSDs cohort was most likely not due to comorbid somatic conditions or microvascular changes.

In contrast to previous findings of pronounced disturbances in the retinal photoreceptor complex in SSDs [15], we found no between-group differences for the central foveal field, the region with the highest cone density, where the inner retinal layers are almost absent [51]. Considering the observed simultaneous thinning of mGCIPL and mRNFL in the parafoveal area, we suspect an underlying process in the inner retinal layers that may involve retinal ganglion cells, synapse formation and neuropil of bipolar cells and retinal ganglion cells, amacrine cells and associated synapses, and horizontal cells [52, 53].

Overall, the effect of SSDs on retinal thickness parameters in our study was rather small (e.g., about 3% reduction in parafoveal MT) but comparable to the 2% reduction in brain volume found in imaging studies [1]. A recent preliminary study indicated an association between outer nuclear layer thinning and smaller total brain and white matter volume and cognitive dysfunction in psychosis probands [12]. However, in the field of retinal investigations in mental illness, there is still a lack of evidence that could reveal, at least in part, the mechanisms underlying the observed retinal alterations. Importantly, also the present study covered only retinal parameters, and it could neither address mechanistical questions nor investigate whether retinal changes are related to altered brain structure or function. Moreover, whether the observed retinal thinning is caused by anterograde or retrograde processes [54] could not be demonstrated in this or previous studies.

Interestingly, our exploratory post hoc analyses revealed a significant association of longer duration of illness with reduced MT measures, although we controlled for multiple covariates, including age. This finding adds to a growing body of evidence suggesting an association between the duration of illness and the extent of retinal changes in SSDs [16]. Alizadeh et al*.* found that a longer duration of illness was negatively associated with several retinal thickness measures in men with chronic SSDs, whereas in acute psychotic stages, a longer duration of illness was associated with higher thickness measures [55]. They suggested that retinal alterations in chronic SSDs could be due to both an acceleration of neurodegeneration and failed neuroregeneration [55].

However, medication effects on retinal structures cannot be ruled out. Further post hoc analyses indicated that higher CPZeq might be associated with reduced parafoveal MT and mRNFL thickness. Importantly, the results of these exploratory analyses were not adjusted for multiple testing and thus must be interpreted with caution; however, an impact of medication on retinal thickness seems plausible, because retinal cells widely express dopamine receptors [56–58] and retinal disturbances are a known adverse effect of some antipsychotics [13, 59]. Nevertheless, higher doses of medication could also reflect more severe disease courses. Of note, neither duration of illness nor medication was associated with pRNFL or mGCIPL thickness, suggesting that even if medication contributes to retinal thinning in SSDs, other factors including disease-specific pathophysiological mechanisms could be involved.

Subsequent analyses showed that a history of treatment with clozapine (lifetime) was significantly associated with lower mean pRNFL thickness but with none of the other parameters studied. Previous or current treatment with clozapine was considered a proxy for treatment resistance, as clozapine is the recommended first-line treatment for treatment-resistant schizophrenia [60, 61]. Whether treatment-resistant SSDs may be characterized by greater pRNFL thinning could be addressed in further studies. Importantly, it is hardly possible to distinguish between medication and disease effects with our cross-sectional study design. Thus, the potential mediator effect of antipsychotic medication needs to be addressed in larger and longitudinal studies with substantial numbers of treatment-naïve first-episode patients.

One important limitation of our OCT investigation is that we used the automated layer segmentation provided by the software of our OCT device, so we were not able to specifically examine the outer retinal layers. Thus, large-scale studies involving the segmentation of all retinal layers would be desirable for future research. Moreover, the number of participants with certain comorbidities such as diabetes was limited. In addition to that, diabetes and hypertension may be underdiagnosed in patients with schizophrenia [13]. Since our study relied on self-report and medical records to assess somatic comorbidities, we may have underestimated the impact of cardiometabolic risk factors on retinal structures. Furthermore, a multivariate analysis does not exclude potential effects of unmeasured confounders, such as chronic stress and various environmental risk factors [62], which could both increase the risk of developing psychosis and could have effects on retinal cells.

Finally, given the exploratory nature of our study, further well-designed studies including studies with a longitudinal design are warranted to replicate our findings and to elucidate the relationship between retina, brain, and clinical parameters in SSDs. Thus, for example replication studies in larger cohorts that systematically measure potential covariates and studies with non-affected relatives of patients with SSDs could be useful to reliably distinguish the effects of confounding environmental factors from directly SSD-driven effects.

Our study provides new evidence for thinning of retinal structures in SSDs. However, the causal mechanisms underlying this association remain to be determined. We suggest that a deeper understanding of the alterations in retinal cytoarchitecture could provide another piece of the puzzle for understanding the pathophysiology of SSDs and that the cost-effective, easy-to-perform method of OCT holds great potential for application in future clinical research.

## Supplementary Information

Below is the link to the electronic supplementary material.

Supplementary file1 (PDF 1922 KB)

## Data Availability

The de-identified individual participant data of this study will be made available upon publication in the Zenodo repository at https://doi.org/10.5281/zenodo.5813675.
