# Supplementary material for: Optical coherence tomography reveals retinal thinning in schizophrenia spectrum disorders
Source: Eur Arch Psychiatry Clin Neurosci. 2022 Aug 5;273(3):575–88. doi: 10.1007/s00406-022-01455-z (PMC10085905; doi:10.1007/s00406-022-01455-z)
Supplement: Supplementary file 1 — Supplementary file1 (PDF 1922 KB) [file 406_2022_1455_MOESM1_ESM.pdf]

Supplemental Information

Optical coherence tomography reveals retinal thinning in schizophrenia spectrum disorders

Emanuel Boudriot[1,*], Benedikt Schworm[2,*], Lenka Krčmář[1,3], Katharina Hanken[1], Iris Jäger[1], Marius Stephan[1,3], Vanessa Gabriel[1], Georgios Ioannou[1], Julian Melcher[1], Genc Hasanaj[1], Mattia Campana[1], Joanna Moussiopoulou[1], Lisa Löhrs[1], Alkomiet Hasan[4], Peter Falkai[1,5], Oliver Pogarell[1], Siegfried Priglinger[2], Daniel Keeser[1,6,7], Christoph Kern[2,*], Elias Wagner[1,*], Florian J. Raabe[1,3,*]

[1]Department of Psychiatry and Psychotherapy, University Hospital, LMU Munich, 80336 Munich, Germany
[2]Department of Ophthalmology, University Hospital, LMU Munich, 80336 Munich, Germany
[3]International Max Planck Research School for Translational Psychiatry (IMPRS-TP), 80804 Munich, Germany
[4]Department of Psychiatry, Psychotherapy and Psychosomatics, Medical Faculty, University of Augsburg, 86156 Augsburg, Germany
[5]Max Planck Institute of Psychiatry, 80804 Munich, Germany
[6]NeuroImaging Core Unit Munich (NICUM), University Hospital, LMU Munich, 80336 Munich, Germany
[7]Munich Center for Neurosciences (MCN), LMU Munich, 82152 Planegg-Martinsried, Germany
*These authors contributed equally.

✉ Florian J. Raabe
florian.raabe@med.uni-muenchen.de

Table of Contents

Supplemental Text 3

Supplemental Figures 4

Fig. S1 | Exemplary illustration of the partial effects of the model predictors on the mean peripapillary retinal nerve fiber layer thickness 4

Fig. S2 | Partial effects of schizophrenia spectrum disorder on retinal thickness and microvasculature, calculated for both eyes (OU) and separately for the right (OD) and left (OS) eyes 5

Fig. S3 | Association of treatment-resistant schizophrenia and retinal thickness 7

Supplemental Tables 8

Table S1 | Estimates for the group coefficients (patients with schizophrenia spectrum disorders vs healthy controls) of the additive models for the right (OD) and left (OS) eyes 8

Table S2 | Association between optical coherence tomography and clinical parameters (oculus uterque) 9

Table S3 | Association of treatment-resistant schizophrenia and retinal thickness 10

Supplemental Model Reports 11

Model Summaries (oculus uterque) 11

Non-linear Effects (oculus uterque) 18

Supplemental Text

After quality control, scans from 153 eyes of 79 patients with schizophrenia spectrum disorders (SSDs) and 166 eyes of 84 healthy controls were eligible for analysis. Of these, 5 patients and 1 healthy control had an intraocular pressure (IOP) greater than or equal to 21 mmHg. One right and 1 left eye in the healthy control group (but none in the patient group) had a spherical equivalent greater than or equal to 6 diopter (D), and 9 right and 9 left eyes of patients and 3 right and 4 left eyes of healthy controls had a spherical equivalent less than or equal to -6 D (if participants had a history of refractive surgery, we used the preoperative values). Furthermore, the following ophthalmological or neurological comorbidities led to exclusion: retinal pigment epithelial detachment (1 control), age-related macular degeneration (1 control, 1 patient with SSD), epiretinal gliosis (1 control), normal-tension glaucoma (1 control), unilateral optic nerve hypoplasia (1 control), history of evisceration of the right eye (1 control), optic disc drusen (1 patient with SSD), intraretinal fluid (1 patient with SSD), central serous chorioretinopathy (1 patient with SSD), presence of fibrae medullares (1 patient with SSD), history of recurrent iritis and cerebral ischemia (1 control), history of encephalitis (2 patients with SSDs) or meningitis (1 control), and history of epilepsy surgery (1 patient with SSD). Note that some participants met more than one exclusion criterion.

Supplemental Figures

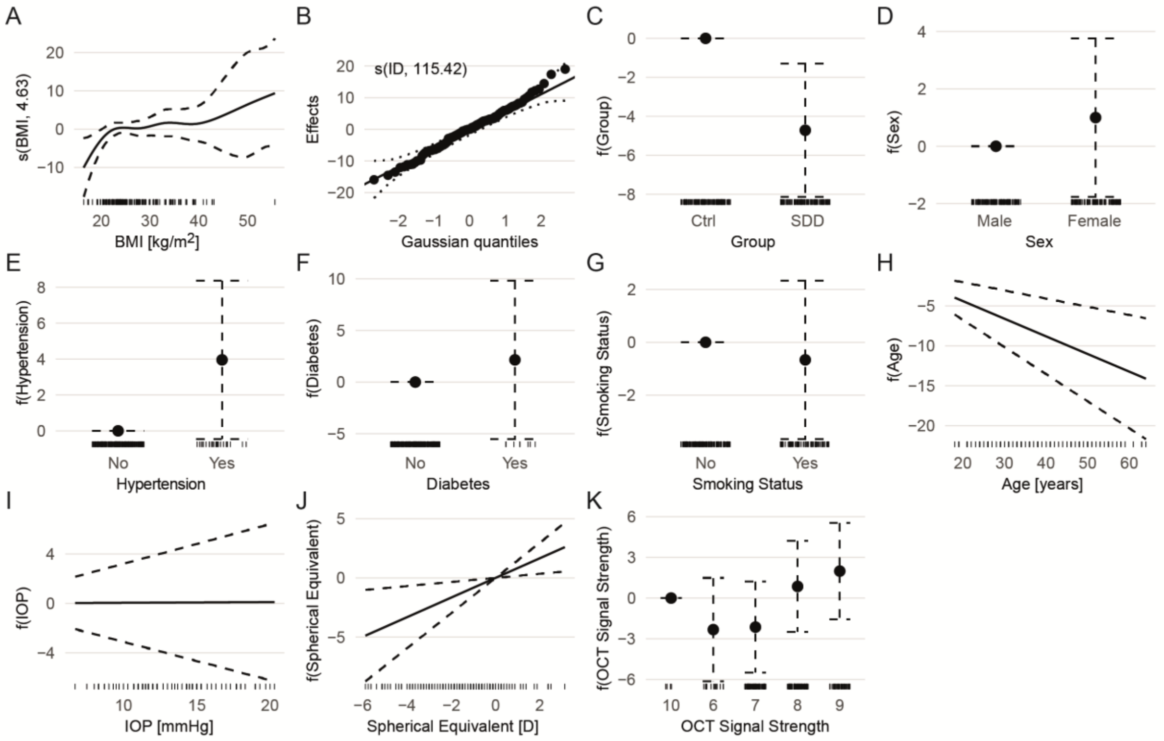

Fig. S1 | Exemplary illustration of the partial effects of the model predictors on the mean peripapillary retinal nerve fiber layer thickness

Shown are **A,** the non-linear effect of the body mass index; **B,** the distribution of the random intercepts; and **C–K,** plots for the parametric coefficients. Dashed lines represent 95% confidence intervals.

Abbreviations: BMI, body mass index; D, diopter; IOP, intraocular pressure; OCT, optical coherence tomography.

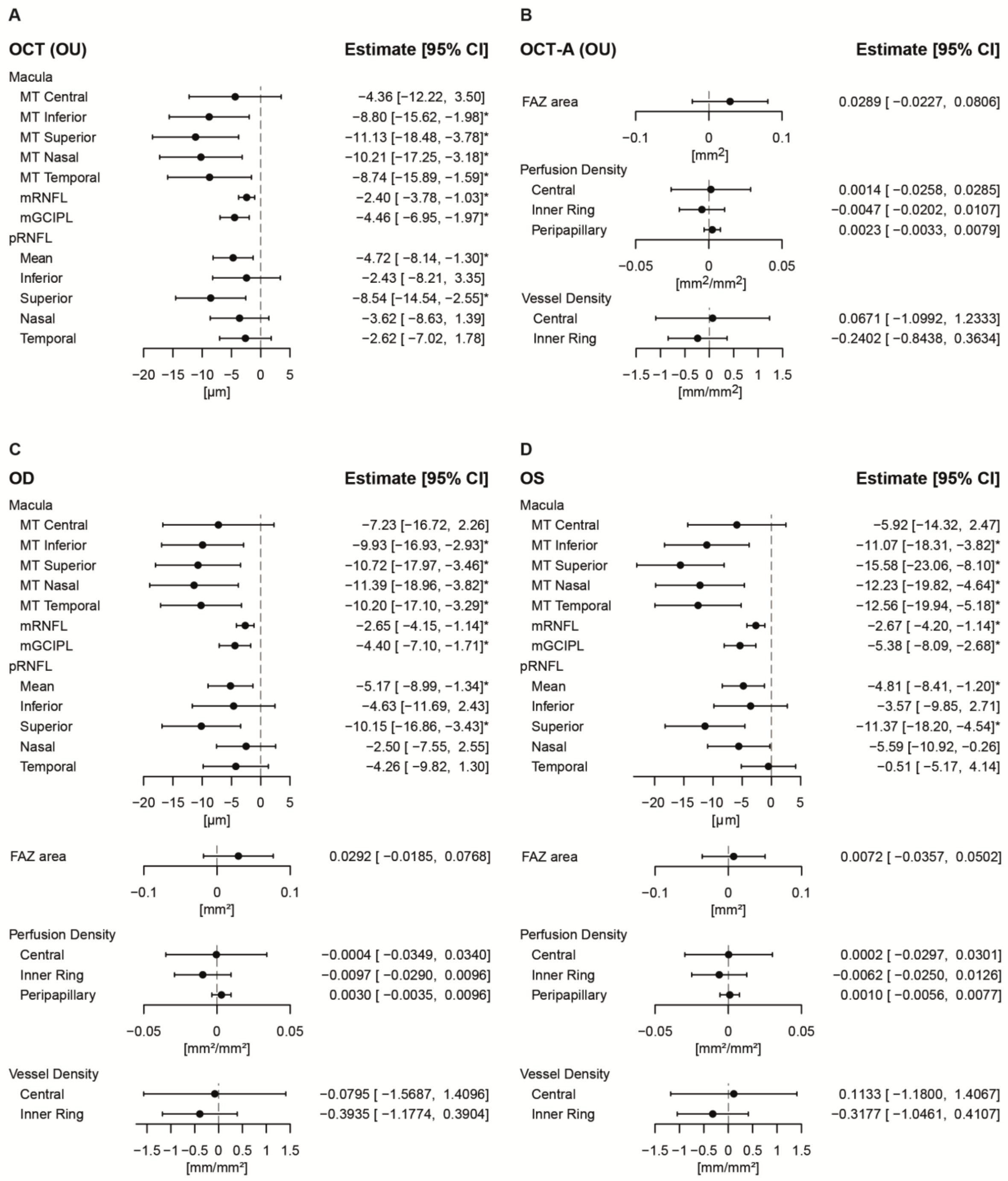

Fig. S2 | Partial effects of schizophrenia spectrum disorder on retinal thickness and microvasculature, calculated for both eyes (OU) and separately for the right (OD) and left (OS) eyes

A Forest plots showing the estimates for the partial effect of the group (patients with schizophrenia spectrum disorders [SSDs] vs healthy controls [Ctrl]) on optical coherence tomography (OCT) parameters, as obtained in additive mixed models. *$p < .05$ (false discovery rate [FDR] adjusted)
B Forest plots showing the estimates for the partial effect of the group (patients with SSDs vs Ctrl) on OCT angiography (OCT-A) parameters, as obtained in additive mixed models. *$p < .05$ (FDR adjusted)
C Forest plots showing the estimates for the partial effect of group (patients with SSDs vs Ctrl) on optical coherence tomography (OCT) and optical coherence tomography angiography (OCT-A) parameters of the right eye (OD), as obtained in additive regression models. *$p < .05$ (FDR adjusted)

D Forest plots showing the estimates for the partial effect of group (SSDs vs Ctrl) on OCT and OCT-A parameters of the left eye (OS), as obtained in additive regression models. *$p < .05$ (FDR adjusted)

Abbreviations: CI, confidence interval; FAZ, foveal avascular zone; mGCIPL, macular ganglion cell–inner plexiform layer; mRNFL, macular retinal nerve fiber layer; MT Central, macular thickness in the central subfield; MT Inferior, macular thickness in the inner inferior subfield; MT Superior, macular thickness in the inner superior subfield; MT Nasal, macular thickness in the inner nasal subfield; MT Temporal, macular thickness in the inner temporal subfield; OD, oculus dexter (right eye); OS, oculus sinister (left eye); OU, oculus uterque (both eyes); pRNFL, peripapillary retinal nerve fiber layer.

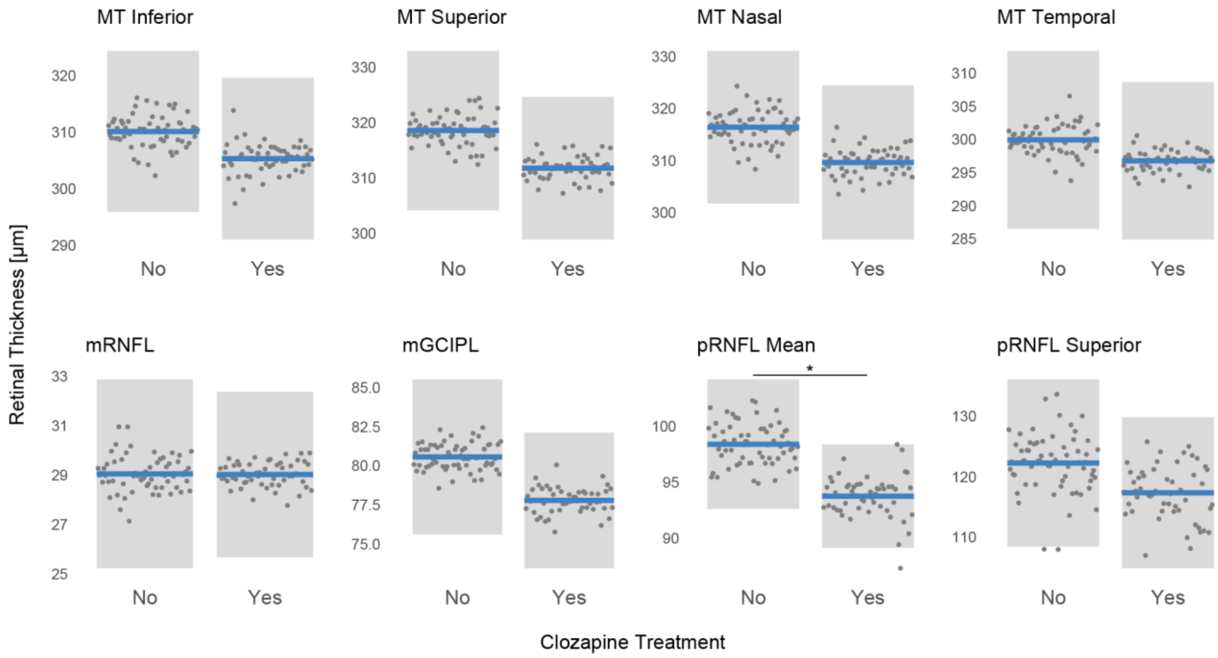

Fig. S3 | Association of treatment-resistant schizophrenia and retinal thickness

Lifetime history of clozapine use (yes) serves as a proxy for treatment resistance. The plots show how the expected values of the outcome variables (blue lines) change as a function of lifetime clozapine use (yes:no) when all other model terms are held fixed. Included are 95% confidence intervals (grey) and dots for the partial residuals. *$p < .05$
Abbreviations: mGCIPL, macular ganglion cell–inner plexiform layer; mRNFL, macular retinal nerve fiber layer; MT Central, macular thickness in the central subfield; MT Inferior, macular thickness in the inner inferior subfield; MT Superior, macular thickness in the inner superior subfield; MT Nasal, macular thickness in the inner nasal subfield; MT Temporal, macular thickness in the inner temporal subfield; pRNFL, peripapillary retinal nerve fiber layer.

Supplemental Tables

Table S1 | Estimates for the group coefficients (patients with schizophrenia spectrum disorders vs healthy controls) of the additive models for the right (OD) and left (OS) eyes

Eye	Dependent OCT/OCT-A Variable	Estimate [95% CI]	n	*p*	*p* (FDR adj.)	
OD	MT, central subfield	-7.2301 [-16.7221, 2.2619]	131	.1382	.2764	*ns*
	MT, inner inferior subfield	-9.9309 [-16.9301, -2.9316]	131	.0063	.0180	*
	MT, inner superior subfield	-10.7172 [-17.9746, -3.4598]	131	.0045	.0153	*
	MT, inner nasal subfield	-11.3905 [-18.9591, -3.8220]	131	.0039	.0149	*
	MT, inner temporal subfield	-10.1959 [-17.0970, -3.2949]	131	.0045	.0153	*
	mRNFL thickness	-2.6471 [-4.1545, -1.1397]	131	.0008	.0079	*
	mGCIPL thickness	-4.4044 [-7.0972, -1.7116]	131	.0018	.0105	*
	pRNFL thickness, mean	-5.1666 [-8.9922, -1.3409]	125	.0094	.0241	*
	pRNFL thickness, inferior	-4.6316 [-11.6925, 2.4294]	125	.2013	.3623	*ns*
	pRNFL thickness, superior	-10.1475 [-16.8638, -3.4311]	125	.0038	.0149	*
	pRNFL thickness, nasal	-2.5045 [-7.5544, 2.5455]	125	.3332	.4735	*ns*
	pRNFL thickness, temporal	-4.2598 [-9.8191, 1.2995]	125	.1360	.2764	*ns*
	FAZ area	0.0292 [-0.0185, 0.0768]	109	.2330	.4059	*ns*
	Perfusion density, central	-0.0004 [-0.0349, 0.0340]	115	.9798	.9883	*ns*
	Perfusion density, inner ring	-0.0097 [-0.0290, 0.0096]	115	.3258	.4735	*ns*
	Vessel density, central	-0.0795 [-1.5687, 1.4096]	115	.9169	.9565	*ns*
	Vessel density, inner ring	-0.3935 [-1.1774, 0.3904]	115	.3276	.4735	*ns*
	Perfusion density, peripapillary	0.0030 [-0.0035, 0.0096]	117	.3664	.5073	*ns*
OS	MT, central subfield	-5.9234 [-14.3157, 2.4689]	132	.1693	.3153	*ns*
	MT, inner inferior subfield	-11.0668 [-18.3098, -3.8239]	132	.0034	.0149	*
	MT, inner superior subfield	-15.5820 [-23.0637, -8.1003]	132	.0001	.0044	*
	MT, inner nasal subfield	-12.2304 [-19.8239, -4.6369]	132	.0020	.0110	*
	MT, inner temporal subfield	-12.5601 [-19.9391, -5.1810]	132	.0011	.0088	*
	mRNFL thickness	-2.6697 [-4.2008, -1.1386]	132	.0009	.0079	*
	mGCIPL thickness	-5.3832 [-8.0860, -2.6805]	132	.0002	.0044	*
	pRNFL thickness, mean	-4.8073 [-8.4144, -1.2001]	131	.0102	.0251	*
	pRNFL thickness, inferior	-3.5678 [-9.8488, 2.7132]	131	.2679	.4307	*ns*
	pRNFL thickness, superior	-11.3731 [-18.2043, -4.5420]	131	.0015	.0099	*
	pRNFL thickness, nasal	-5.5925 [-10.9207, -0.2643]	131	.0420	.0907	*ns*
	pRNFL thickness, temporal	-0.5109 [-5.1655, 4.1437]	131	.8300	.9338	*ns*
	FAZ area	0.0072 [-0.0357, 0.0502]	111	.7418	.8708	*ns*
	Perfusion density, central	0.0002 [-0.0297, 0.0301]	116	.9883	.9883	*ns*
	Perfusion density, inner ring	-0.0062 [-0.0250, 0.0126]	116	.5199	.6380	*ns*
	Vessel density, central	0.1133 [-1.1800, 1.4067]	116	.8640	.9521	*ns*
	Vessel density, inner ring	-0.3177 [-1.0461, 0.4107]	116	.3946	.5328	*ns*
	Perfusion density, peripapillary	0.0010 [-0.0056, 0.0077]	114	.7678	.8821	*ns*

Abbreviations: FAZ, foveal avascular zone; MT, macular thickness; mRNFL, macular retinal nerve fiber layer; mGCIPL, macular combined ganglion cell layer and inner plexiform layer; n, number of eyes (patients with schizophrenia spectrum disorders and healthy controls); ns, not significant; OCT, optical coherence tomography; OCT-A, optical coherence tomography angiography; OD, oculus dexter; OS, oculus sinister; *p*, *p* value; *p* (FDR adj.), false discovery rate adjusted *p* value; pRNFL, peripapillary retinal nerve fiber layer.
$^{*}p < .05$

Table S2 | Association between optical coherence tomography and clinical parameters (oculus uterque)

Clinical parameter	Dependent OCT measure	Estimate [95% CI]	N	n	*p*	
Chlorpromazine equivalent doses[a]	MT, inner inferior subfield	-0.0144 [-0.0282, -0.0006]	54	101	.0468	*
	MT, inner superior subfield	-0.0125 [-0.0266, 0.0016]	54	101	.0889	*ns*
	MT, inner nasal subfield	-0.0186 [-0.0318, -0.0053]	54	101	.0087	*
	MT, inner temporal subfield	-0.0169 [-0.0302, -0.0037]	54	101	.0162	*
	mRNFL	-0.0029 [-0.0052, -0.0005]	54	101	.0199	*
	mGCIPL	-0.0011 [-0.0072, 0.0050]	54	101	.7307	*ns*
	pRNFL, mean	0.0019 [-0.0062, 0.0099]	54	101	.6522	*ns*
	pRNFL, superior	-0.0001 [-0.0129, 0.0128]	54	101	.9924	*ns*
Duration of illness[b]	MT, inner inferior subfield	-0.7078 [-1.2977, -0.1180]	63	119	.0224	*
	MT, inner superior subfield	-0.8307 [-1.4156, -0.2458]	63	119	.0075	*
	MT, inner nasal subfield	-0.6398 [-1.2503, -0.0292]	63	119	.0449	*
	MT, inner temporal subfield	-0.7085 [-1.3297, -0.0874]	63	119	.0301	*
	mRNFL	-0.1729 [-0.3025, -0.0432]	63	119	.0116	*
	mGCIPL	-0.2526 [-0.5182, 0.0130]	63	119	.0686	*ns*
	pRNFL, mean	-0.0284 [-0.2968, 0.2401]	62	117	.8367	*ns*
	pRNFL, superior	0.0828 [-0.4259, 0.5915]	62	117	.7509	*ns*

Abbreviations: CI, confidence interval; mGCIPL, macular ganglion cell–inner plexiform layer; mRNFL, macular retinal nerve fiber layer; MT, macular thickness; N, number of patients; n, number of eyes; ns, not significant; *p*, *p* value; pRNFL, peripapillary retinal nerve fiber layer.

[a]Ten patients had missing data for chlorpromazine equivalent doses.

[b]Two patients had missing data for duration of illness.

*$p < .05$

Table S3 | Association of treatment-resistant schizophrenia and retinal thickness

Dependent OCT measure	Estimate [95% CI]	N	n	*p*	
MT, inner inferior subfield	-4.8176 [-13.4839, 3.8488]	63	119	.2808	*ns*
MT, inner superior subfield	-6.7795 [-15.5244, 1.9653]	63	119	.1354	*ns*
MT, inner nasal subfield	-6.7771 [-15.6789, 2.1248]	63	119	.1416	*ns*
MT, inner temporal subfield	-3.1537 [-11.6526, 5.3451]	63	119	.4706	*ns*
mRNFL thickness	-0.0280 [-1.7476, 1.6915]	63	119	.9746	*ns*
mGCIPL thickness	-2.7772 [-5.8963, 0.3419]	63	119	.0869	*ns*
pRNFL thickness, mean	-4.6366 [-8.1235, -1.1497]	62	118	.0117	*
pRNFL thickness, superior	-4.9276 [-11.1631, 1.3079]	62	118	.1269	*ns*

Lifetime history of treatment with clozapine (yes:no) serves as a proxy for treatment resistance. Information on clozapine use was missing in two patients.

Abbreviations: CI, confidence interval; mGCIPL, macular ganglion cell–inner plexiform layer; mRNFL, macular retinal nerve fiber layer; MT, macular thickness; N, number of patients; n, number of eyes; ns, not significant; *p*, *p* value; pRNFL, peripapillary retinal nerve fiber layer.
*p < .05

Supplemental Model Reports

Model Summaries (oculus uterque)

Abbreviations: BMI, body mass index; edf, estimated degrees of freedom; FAZ, foveal avascular zone; ID, random intercept for the participant identification; IOP, intraocular pressure; mGCIPL, macular ganglion cell–inner plexiform layer; mRNFL, macular retinal nerve fiber layer; OCT, optical coherence tomography; pRNFL, peripapillary retinal nerve fiber layer; Ref.df, reference degrees of freedom; Std. Error, Standard error.

**A. Parametric coefficients**	**Estimate**	**Std. Error**	***t* value**	***p* value**
Intercept	273.2090	4.5446	6.1168	< .0001
Group	-4.3611	4.0119	-1.0870	.2792
Sex (female)	-17.2888	3.2214	-5.3669	< .0001
Hypertension	17.2290	5.1172	3.3669	.0010
Diabetes	-5.3662	8.0982	-0.6626	.5088
Smoking	3.7847	3.4822	1.0868	.2792
IOP	-0.3476	0.2581	-1.3470	.1805
OCT signal strength (9)	0.3805	1.0108	0.3765	.7072
OCT signal strength (8)	2.2587	1.1700	1.9306	.0558
OCT signal strength (7)	4.1718	1.3616	3.0639	.0027
OCT signal strength (6)	0.2940	3.6488	0.0806	.9359
**B. Smooth terms**	**edf**	**Ref.df**	***F* value**	***p* value**
Age	1.5389	1.5488	4.1404	.0528
BMI	2.7786	2.7960	5.1438	.0024
Spherical equivalent	3.0433	3.7283	1.2313	.2414
ID	121.5032	128.0000	42.1426	< .0001
**Dependent: Macular Thickness [µm], Central Subfield**				

**A. Parametric coefficients**	**Estimate**	**Std. Error**	***t* value**	***p* value**
Intercept	334.3737	5.8677	56.9855	< .0001
Group	-8.8016	3.4786	-2.5302	.0127
Sex (female)	-11.8585	2.7802	-4.2653	< .0001
Hypertension	7.0026	4.4419	1.5765	.1175
Diabetes	-2.2719	6.9312	-0.3278	.7436
Smoking	3.4917	3.0136	1.1586	.2488
Age	-0.1229	0.1194	-1.0293	.3053
IOP	-0.1325	0.2568	-0.5159	.6068
OCT Signal Strength (9)	0.2792	1.0469	0.2667	.7901
OCT Signal Strength (8)	1.2895	1.2076	1.0678	.2877
OCT Signal Strength (7)	4.1857	1.4047	2.9798	.0035
OCT Signal Strength (6)	1.6825	3.7213	0.4521	.6520
**B. Smooth terms**	**edf**	**Ref.df**	***F* value**	***p* value**
BMI	6.0128	6.0318	1.6420	.1426
Spherical Equivalent	4.1455	4.9212	1.0004	.3885
ID	116.9291	128.0000	25.7504	< .0001
**Dependent: Macular Thickness [µm], Inner Inferior Subfield**				

A. Parametric coefficients	Estimate	Std. Error	*t* value	*p* value
Intercept	334.8099	4.4465	75.2981	< .0001
Group	-11.1310	3.7503	-2.9680	.0036
Sex (female)	-10.5523	3.0106	-3.5050	.0006
Hypertension	8.7140	4.7715	1.8263	.0702
Diabetes	-6.0631	7.5278	-0.8054	.4221
Smoking	3.5736	3.2398	1.1030	.2721
IOP	-0.2393	0.2589	-0.9240	.3573
OCT Signal Strength (9)	0.9599	1.0314	0.9306	.3538
OCT Signal Strength (8)	2.3848	1.1923	2.0002	.0477
OCT Signal Strength (7)	4.9523	1.3897	3.5636	.0005
OCT Signal Strength (6)	1.4085	3.7104	0.3796	.7049
**B. Smooth terms**	**edf**	**Ref.df**	***F* value**	***p* value**
Age	1.0000	1.0000	0.4016	.5274
BMI	3.5992	3.6209	1.2130	.2433
Spherical Equivalent	2.3787	2.9159	0.9141	.4735
ID	120.5127	128.0000	28.1676	< .0001
**Dependent: Macular Thickness [µm], Inner Superior Subfield**				

A. Parametric coefficients	Estimate	Std. Error	*t* value	*p* value
Intercept	340.0058	5.8910	57.7165	< .0001
Group	-10.2115	3.5898	-2.8446	.0052
Sex (female)	-14.1124	2.8825	-4.8960	< .0001
Hypertension	9.9115	4.6050	2.1523	.0333
Diabetes	-4.5759	7.1699	-0.6382	.5245
Smoking	4.6525	3.1207	1.4908	.1385
Age	0.0018	0.1227	0.0144	.9885
IOP	-0.3504	0.2473	-1.4169	.1590
Spherical Equivalent	0.1148	0.5780	0.1987	.8428
OCT Signal Strength (9)	-0.3001	0.9851	-0.3047	.7611
OCT Signal Strength (8)	1.4621	1.1363	1.2867	.2006
OCT Signal Strength (7)	3.5293	1.3285	2.6565	.0089
OCT Signal Strength (6)	-0.5568	3.5623	-0.1563	.8760
**B. Smooth terms**	**edf**	**Ref.df**	***F* value**	***p* value**
BMI	6.0564	6.0718	1.9368	.0789
ID	118.3326	128.0000	32.1257	< .0001
**Dependent: Macular Thickness [µm], Inner Nasal Subfield**				

A. Parametric coefficients	Estimate	Std. Error	*t* value	*p* value
Intercept	319.9348	5.8473	54.7148	< .0001
Group	-8.7382	3.6490	-2.3947	.0181
Sex (female)	-11.9310	2.9389	-4.0597	.0001
Hypertension	9.4892	4.6584	2.0370	.0438
Diabetes	-5.2826	7.3425	-0.7195	.4732
Smoking	3.3686	3.1605	1.0658	.2886
Age	0.0358	0.1260	0.2843	.7767
IOP	-0.2508	0.2267	-1.1064	.2707
OCT Signal Strength (9)	0.7056	0.8801	0.8017	.4242
OCT Signal Strength (8)	2.3049	1.0187	2.2625	.0254
OCT Signal Strength (7)	5.5012	1.1882	4.6300	< .0001
OCT Signal Strength (6)	3.4695	3.1971	1.0852	.2799
**B. Smooth terms**	**edf**	**Ref.df**	***F* value**	***p* value**
BMI	3.5786	3.5952	1.1014	.3259
Spherical Equivalent	1.7856	2.1944	0.6364	.5194
ID	121.7209	128.0000	38.4858	< .0001
**Dependent: Macular Thickness [µm], Inner Temporal Subfield**				

A. Parametric coefficients	Estimate	Std. Error	*t* value	*p* value
Intercept	34.0468	1.3612	25.0119	< .0001
Group	-2.4031	0.7006	-3.4302	.0008
Sex (female)	-0.9940	0.5433	-1.8295	.0696
Hypertension	2.6332	0.9035	2.9143	.0042
Diabetes	0.3703	1.4525	0.2549	.7992
Smoking	0.1294	0.6175	0.2095	.8344
BMI	0.0498	0.0531	0.9381	.3500
OCT Signal Strength (9)	-0.4582	0.2515	-1.8215	.0709
OCT Signal Strength (8)	-0.4938	0.2893	-1.7067	.0903
OCT Signal Strength (7)	-0.5731	0.3381	-1.6948	.0925
OCT Signal Strength (6)	0.0608	0.8889	0.0684	.9456
**B. Smooth terms**	**edf**	**Ref.df**	***F* value**	***p* value**
Age	2.7193	2.7534	1.3709	.3559
IOP	1.0000	1.0000	2.1981	.1406
Spherical Equivalent	1.9620	2.3446	10.8054	< .0001
ID	118.4307	128.0000	20.1202	< .0001
**Dependent: mRNFL Thickness [µm]**				

A. Parametric coefficients	Estimate	Std. Error	*t* value	*p* value
Intercept	90.1545	2.0001	45.0739	< .0001
Group	-4.4632	1.2698	-3.5148	.0006
Sex (female)	-1.2838	1.0233	-1.2546	.2120
Hypertension	1.1054	1.6267	0.6795	.4981
Diabetes	1.6679	2.5479	0.6546	.5139
Smoking	-0.5392	1.1056	-0.4876	.6267
Age	-0.1160	0.0436	-2.6576	.0089
IOP	-0.0366	0.0755	-0.4853	.6283
Spherical Equivalent	0.3831	0.1840	2.0821	.0394
OCT Signal Strength (9)	0.1794	0.2907	0.6173	.5381
OCT Signal Strength (8)	0.2992	0.3361	0.8903	.3750
OCT Signal Strength (7)	0.8009	0.3926	2.0402	.0434
OCT Signal Strength (6)	-0.2134	1.0620	-0.2009	.8411
**B. Smooth terms**	**edf**	**Ref.df**	***F* value**	***p* value**
BMI	5.1265	5.1400	2.6407	.0354
ID	120.6416	129.0000	46.5600	< .0001
**Dependent: mGCIPL Thickness [µm]**				

A. Parametric coefficients	Estimate	Std. Error	*t* value	*p* value
Intercept	103.4690	3.6427	28.4041	< .0001
Group	-4.7171	1.7441	-2.7046	.0078
Sex (female)	0.9984	1.4096	0.7083	.4801
Hypertension	3.9553	2.2526	1.7559	.0816
Diabetes	2.1524	3.9156	0.5497	.5835
Smoking	-0.6641	1.5304	-0.4339	.6651
Age	-0.2205	0.0603	-3.6580	.0004
IOP	0.0053	0.1611	0.0328	.9739
Spherical Equivalent	0.8312	0.3356	2.4766	.0146
OCT Signal Strength (9)	1.9862	1.8147	1.0945	.2759
OCT Signal Strength (8)	0.8564	1.7105	0.5007	.6175
OCT Signal Strength (7)	-2.1397	1.7168	-1.2463	.2150
OCT Signal Strength (6)	-2.3242	1.9447	-1.1951	.2343
**B. Smooth terms**	**edf**	**Ref.df**	***F* value**	***p* value**
BMI	4.6284	4.6693	1.8594	.1390
ID	115.4244	128.0000	13.7528	< .0001
**Dependent: pRNFL Thickness [µm], Mean** (see also **Fig. S1**)				

A. Parametric coefficients	Estimate	Std. Error	*t* value	*p* value
Intercept	137.6984	5.2765	26.0968	< .0001
Group	-2.4424	2.9302	-0.8335	.4061
Sex (female)	3.7009	2.3551	1.5715	.1186
Hypertension	2.2986	3.7693	0.6098	.5431
Diabetes	2.0951	6.5630	0.3192	.7501
Smoking	-2.5910	2.5447	-1.0182	.3105
Age	-0.3336	0.1013	-3.2924	.0013
Spherical Equivalent	3.0393	0.6024	5.0453	< .0001
OCT Signal Strength (9)	3.2507	3.8028	0.8548	.3943
OCT Signal Strength (8)	-1.1920	3.5974	-0.3313	.7409
OCT Signal Strength (7)	-5.0012	3.6210	-1.3812	.1697
OCT Signal Strength (6)	-2.9702	4.0715	-0.7295	.4670
**B. Smooth terms**	**edf**	**Ref.df**	***F* value**	***p* value**
BMI	3.1300	3.1955	1.5405	.2023
IOP	3.1616	3.7742	0.9764	.4976
ID	111.4267	128.0000	9.4644	< .0001
**Dependent: pRNFL Thickness [µm], Inferior Quadrant**				

A. Parametric coefficients	Estimate	Std. Error	*t* value	*p* value
Intercept	111.6996	8.5468	13.0691	< .0001
Group	-8.5430	3.0592	-2.7926	.0060
Sex (female)	-2.2556	2.3622	-0.9548	.3414
Hypertension	3.6689	3.9994	0.9174	.3606
Diabetes	6.1390	6.9107	0.8883	.3759
Smoking	-0.7462	2.6828	-0.2781	.7813
BMI	0.5734	0.2322	2.4692	.0148
IOP	-0.4265	0.3546	-1.2027	.2312
Spherical Equivalent	1.0921	0.6713	1.6270	.1061
OCT Signal Strength (9)	1.6070	4.9052	0.3276	.7437
OCT Signal Strength (8)	2.7285	4.6574	0.5858	.5590
OCT Signal Strength (7)	0.4210	4.6763	0.0900	.9284
OCT Signal Strength (6)	0.3800	5.2525	0.0723	.9424
**B. Smooth terms**	**edf**	**Ref.df**	***F* value**	***p* value**
Age	1.4669	1.5169	7.5300	.0016
ID	106.4008	128.0000	5.5478	< .0001
**Dependent: pRNFL Thickness [µm], Superior Quadrant**				

A. Parametric coefficients	Estimate	Std. Error	*t* value	*p* value
Intercept	73.9080	4.7490	15.5629	< .0001
Group	-3.6164	2.5557	-1.4150	.1595
Sex (female)	-0.0272	2.0339	-0.0134	.9894
Hypertension	4.3126	3.2875	1.3118	.1919
Diabetes	3.5385	5.7281	0.6177	.5379
Smoking	0.3619	2.2112	0.1637	.8702
Age	-0.0615	0.0885	-0.6954	.4881
Spherical Equivalent	0.6798	0.5359	1.2684	.2070
OCT Signal Strength (9)	0.9975	3.5743	0.2791	.7806
OCT Signal Strength (8)	1.1439	3.3873	0.3377	.7361
OCT Signal Strength (7)	-3.3302	3.4072	-0.9774	.3302
OCT Signal Strength (6)	-6.6669	3.8279	-1.7417	.0840
**B. Smooth terms**	**edf**	**Ref.df**	***F* value**	***p* value**
BMI	2.3809	2.4519	1.3388	.1716
IOP	4.7879	5.4914	2.2666	.0465
ID	109.6315	128.0000	6.4177	< .0001
**Dependent: pRNFL Thickness [µm], Nasal Quadrant**				

A. Parametric coefficients	Estimate	Std. Error	*t* value	*p* value
Intercept	66.2877	4.7178	14.0504	< .0001
Group	-2.6159	2.2445	-1.1655	.2460
Sex (female)	1.3230	1.7567	0.7531	.4527
Hypertension	4.3288	2.9249	1.4800	.1413
Diabetes	-4.7652	5.0781	-0.9384	.3498
Smoking	-2.0171	1.9808	-1.0183	.3104
IOP	-0.0819	0.2458	-0.3330	.7396
Spherical Equivalent	-0.9353	0.4773	-1.9596	.0522
OCT Signal Strength (9)	2.3606	3.1696	0.7448	.4578
OCT Signal Strength (8)	0.4201	3.0010	0.1400	.8889
OCT Signal Strength (7)	-1.2443	3.0121	-0.4131	.6802
OCT Signal Strength (6)	0.1408	3.3939	0.0415	.9670
**B. Smooth terms**	**edf**	**Ref.df**	***F* value**	***p* value**
Age	2.0561	2.1128	1.8793	.1427
BMI	1.2570	1.2825	0.2862	.5217
ID	110.4824	128.0000	8.1696	< .0001
**Dependent: pRNFL Thickness [µm], Temporal Quadrant**				

A. Parametric coefficients	Estimate	Std. Error	*t* value	*p* value
Intercept	0.1837	0.0566	3.2483	.0016
Group	0.0289	0.0263	1.0994	.2743
Sex (female)	0.0326	0.0206	1.5843	.1164
Hypertension	-0.0273	0.0326	-0.8369	.4047
Diabetes	-0.0320	0.0566	-0.5649	.5735
Smoking	0.0074	0.0235	0.3169	.7520
BMI	-0.0018	0.0020	-0.8810	.3805
IOP	0.0034	0.0021	1.6138	.1098
OCT Signal Strength (9)	0.0047	0.0066	0.7120	.4782
OCT Signal Strength (8)	0.0002	0.0077	0.0252	.9800
**B. Smooth terms**	**edf**	**Ref.df**	***F* value**	***p* value**
Age	6.3031	6.3383	1.1583	.3261
Spherical Equivalent	6.4516	7.1196	1.1888	.2727
ID	100.2436	111.0000	17.3688	< .0001
**Dependent: FAZ size [mm^2]**				

A. Parametric coefficients	Estimate	Std. Error	*t* value	*p* value
Intercept	0.2177	0.0293	7.4221	< .0001
Group	0.0014	0.0139	0.0993	.9211
Sex (female)	-0.0394	0.0107	-3.6876	.0003
Hypertension	0.0214	0.0167	1.2849	.2011
Diabetes	0.0308	0.0287	1.0742	.2846
Smoking	-0.0064	0.0114	-0.5591	.5770
Age	0.0006	0.0005	1.3237	.1879
IOP	0.0020	0.0017	1.1806	.2399
OCT Signal Strength (9)	-0.0332	0.0083	-4.0221	.0001
OCT Signal Strength (8)	-0.0940	0.0094	-10.0272	< .0001
**B. Smooth terms**	**edf**	**Ref.df**	***F* value**	***p* value**
BMI	3.7605	3.9446	4.6534	.0014
Spherical Equivalent	3.4900	3.9468	0.7083	.5090
ID	79.3434	117.0000	2.5772	< .0001
**Dependent: Perfusion Density [mm^2/mm^2], Macula, Central Subfield**				

A. Parametric coefficients	Estimate	Std. Error	*t* value	*p* value
Intercept	0.4485	0.0071	63.2070	< .0001
Group	-0.0047	0.0079	-0.6010	.5487
Sex (female)	-0.0114	0.0061	-1.8580	.0649
Hypertension	0.0053	0.0095	0.5550	.5796
Diabetes	0.0069	0.0165	0.4186	.6760
Smoking	-0.0065	0.0066	-0.9893	.3239
OCT Signal Strength (9)	-0.0195	0.0060	-3.2239	.0015
OCT Signal Strength (8)	-0.0677	0.0067	-10.0685	< .0001
**B. Smooth terms**	**edf**	**Ref.df**	***F* value**	***p* value**
Age	2.0139	2.2909	1.9305	.1235
BMI	3.6046	3.9874	1.1036	.3426
IOP	2.4464	2.8725	0.9923	.3239
Spherical Equivalent	3.7308	4.2684	1.8390	.1053
ID	44.7404	117.0000	0.6458	.0007

Dependent: Perfusion Density [mm^2/mm^2], Macula, Parafoveal Ring

A. Parametric coefficients	Estimate	Std. Error	*t* value	*p* value
Intercept	9.5611	1.2614	7.5796	< .0001
Group	0.0671	0.5950	0.1127	.9104
Sex (female)	-1.7207	0.4596	-3.7436	.0003
Hypertension	0.9162	0.7177	1.2764	.2040
Diabetes	1.2819	1.2339	1.0389	.3007
Smoking	-0.3491	0.4916	-0.7102	.4788
Age	0.0259	0.0210	1.2357	.2187
IOP	0.0942	0.0714	1.3194	.1893
OCT Signal Strength (9)	-1.3854	0.3563	-3.8882	.0002
OCT Signal Strength (8)	-3.9175	0.4043	-9.6896	< .0001
**B. Smooth terms**	**edf**	**Ref.df**	***F* value**	***p* value**
BMI	4.3229	4.5085	4.9530	.0010
Spherical Equivalent	3.5355	3.9976	0.8339	.4885
ID	78.4464	117.0000	2.5303	< .0001

Dependent: Vessel Density [mm/mm^2], Macula, Central Subfield

A. Parametric coefficients	Estimate	Std. Error	*t* value	*p* value
Intercept	18.4958	0.2794	66.1942	< .0001
Group	-0.2402	0.3079	-0.7801	.4364
Sex (female)	-0.3809	0.2388	-1.5954	.1124
Hypertension	0.3023	0.3715	0.8137	.4169
Diabetes	0.3495	0.6455	0.5414	.5889
Smoking	-0.2682	0.2573	-1.0425	.2987
OCT Signal Strength (9)	-0.7870	0.2431	-3.2368	.0014
OCT Signal Strength (8)	-2.6877	0.2696	-9.9706	< .0001
**B. Smooth terms**	**edf**	**Ref.df**	***F* value**	***p* value**
Age	2.0574	2.3694	2.1428	.0915
BMI	3.6913	4.1180	1.1750	.3535
IOP	2.3757	2.8044	0.8422	.3755
Spherical Equivalent	3.8080	4.3781	1.5343	.1571
ID	37.7865	117.0000	0.4916	.0047

Dependent: Vessel Density [mm/mm^2], Macula, Parafoveal Ring

**A. Parametric coefficients**	**Estimate**	**Std. Error**	***t* value**	***p* value**
Intercept	0.4518	0.0060	75.1220	< .0001
Group	0.0023	0.0029	0.8074	.4210
Sex (female)	0.0066	0.0023	2.8695	.0048
Hypertension	0.0004	0.0037	0.1110	.9118
Diabetes	0.0090	0.0069	1.2933	.1983
Smoking	-0.0002	0.0001	-2.1553	.0331
Age	0.0001	0.0003	0.3864	.6998
IOP	0.0006	0.0006	0.8836	.3786
Spherical Equivalent	-0.0015	0.0025	-0.6076	.5446
OCT Signal Strength (9)	-0.0030	0.0015	-1.9982	.0479
OCT Signal Strength (8)	-0.0046	0.0015	-3.0018	.0032
**B. Smooth terms**	**edf**	**Ref.df**	***F* value**	***p* value**
BMI	3.9110	4.0333	1.3377	.2697
ID	91.4338	117.0000	4.5949	< .0001

Dependent: Perfusion Density [mm^2/mm^2], Peripapillary

Non-linear Effects (oculus uterque)

Abbreviations: BMI, body mass index; FAZ, foveal avascular zone; ID, random intercept for the participant identification; IOP, intraocular pressure; mGCIPL, macular ganglion cell–inner plexiform layer; mRNFL, macular retinal nerve fiber layer; pRNFL, peripapillary retinal nerve fiber layer; SphE, Spherical Equivalent.

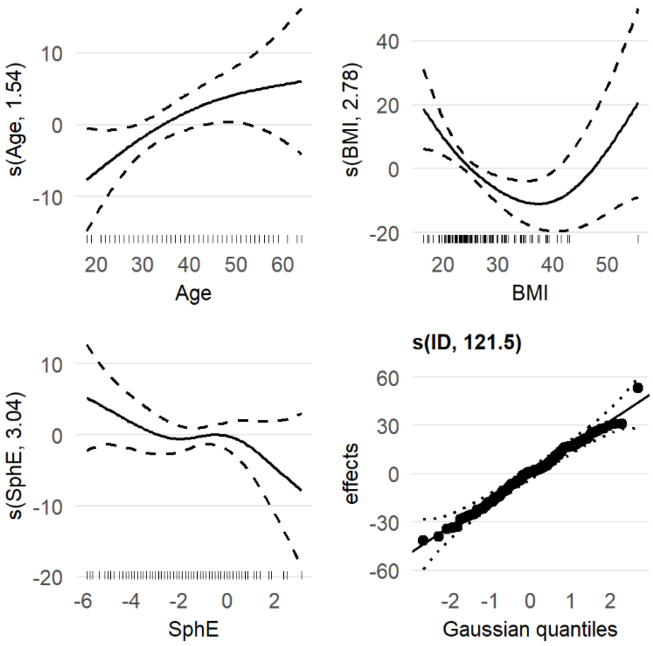

Dependent: Macular Thickness [µm], Central Subfield

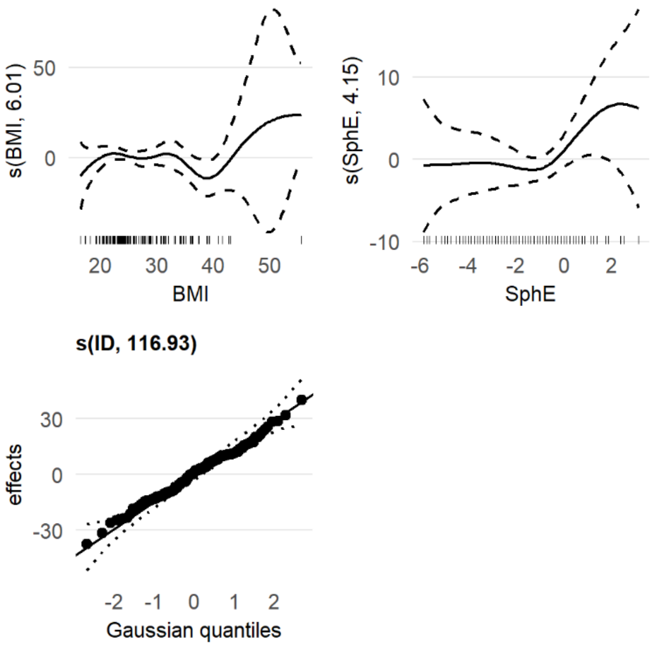

Dependent: Macular Thickness [µm], Inner Inferior Subfield

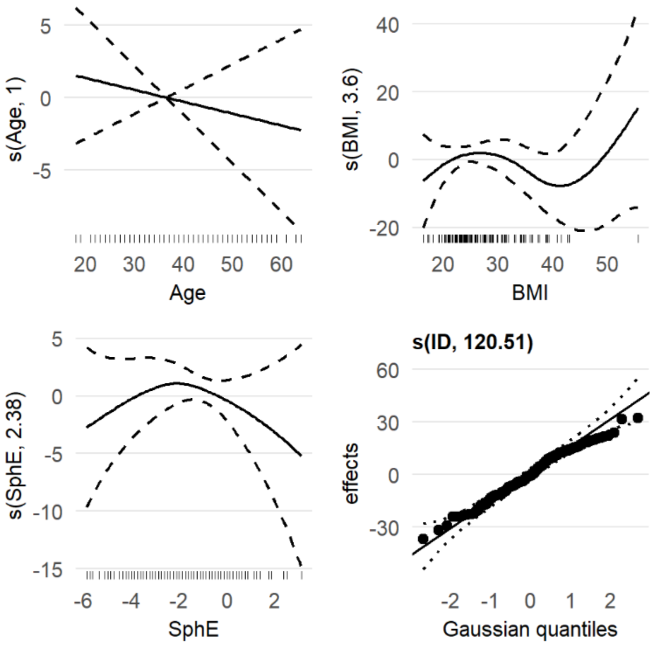

Dependent: Macular Thickness [µm], Inner Superior Subfield

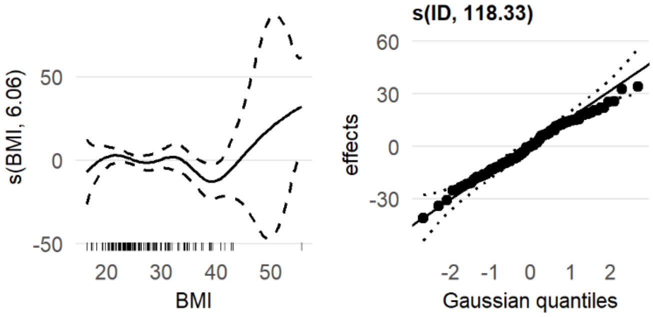

Dependent: Macular Thickness [µm], Inner Nasal Subfield

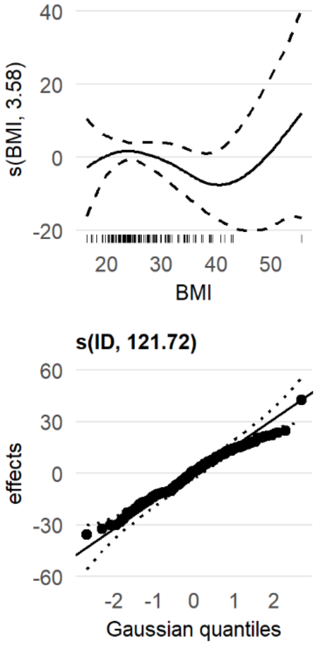

Dependent: Macular Thickness [µm], Inner Temporal Subfield

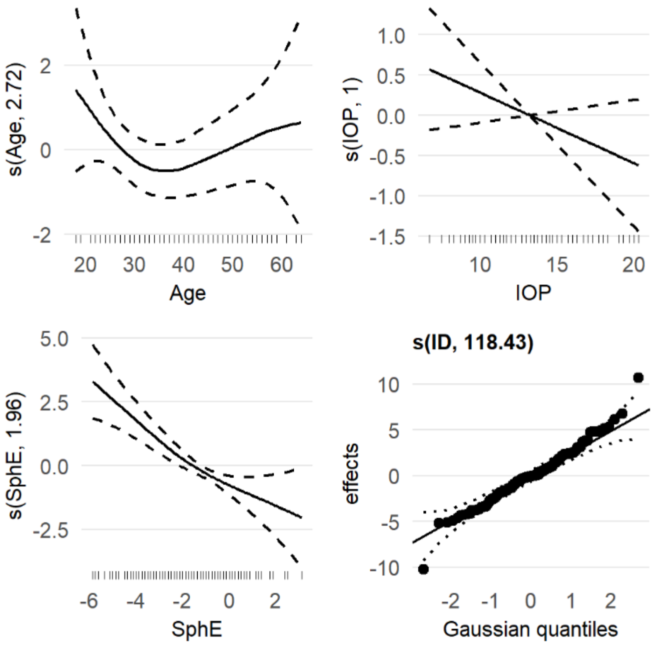

Dependent: mRNFL Thickness [µm]

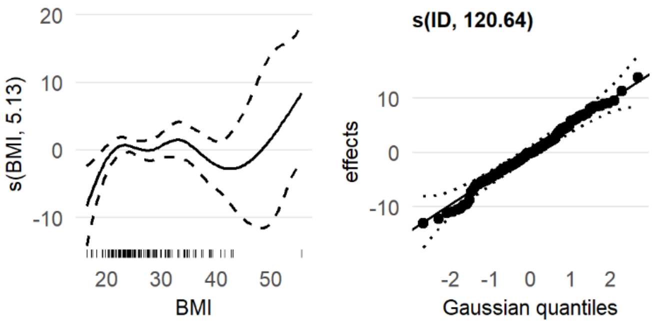

Dependent: mGCIPL Thickness [µm]

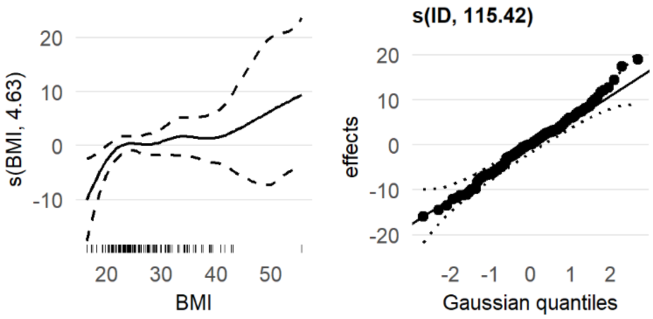

Dependent: pRNFL Thickness [µm], Mean

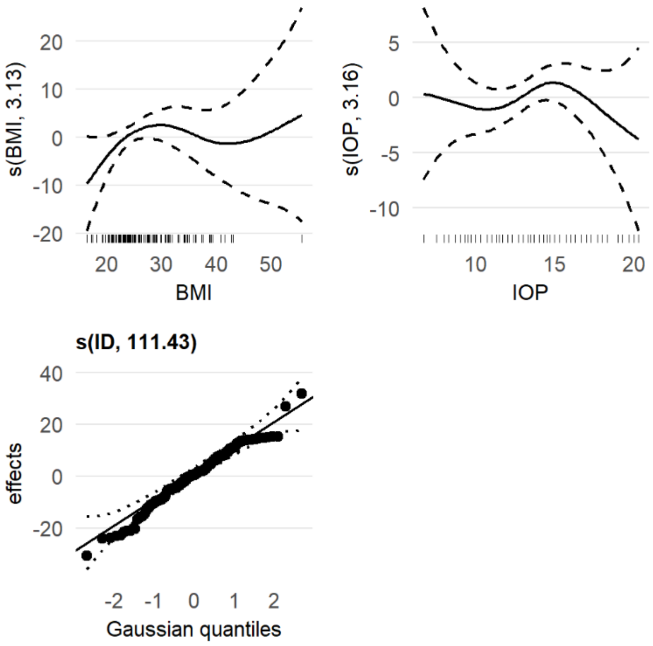

Dependent: pRNFL Thickness [µm], Inferior Quadrant

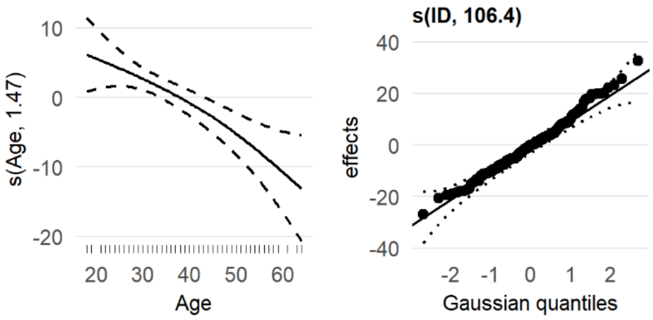

Dependent: pRNFL Thickness [µm], Superior Quadrant

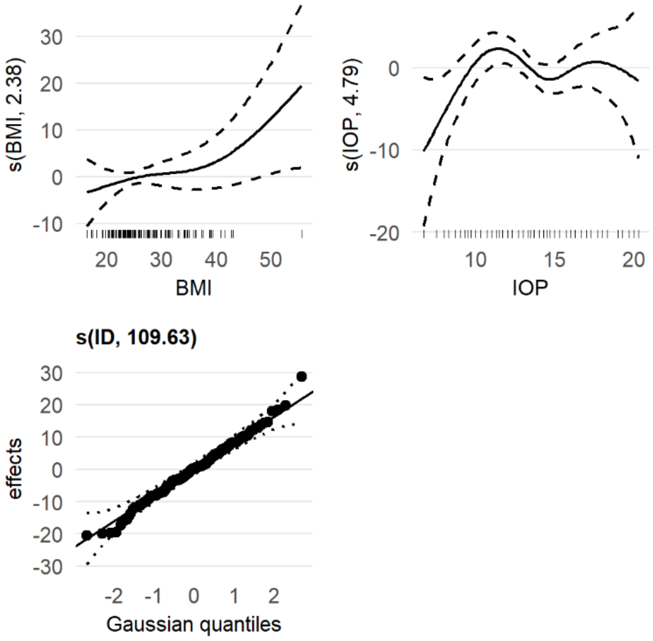

Dependent: pRNFL Thickness [µm], Nasal Quadrant

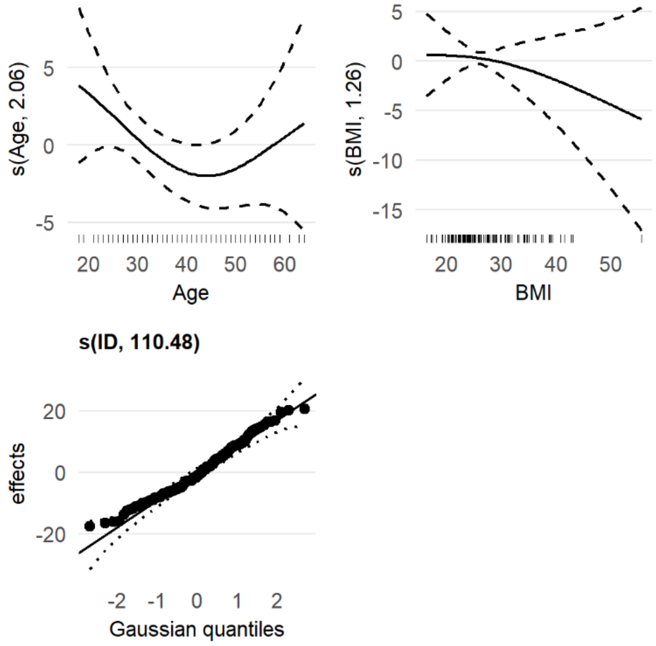

Dependent: pRNFL Thickness [µm], Temporal Quadrant

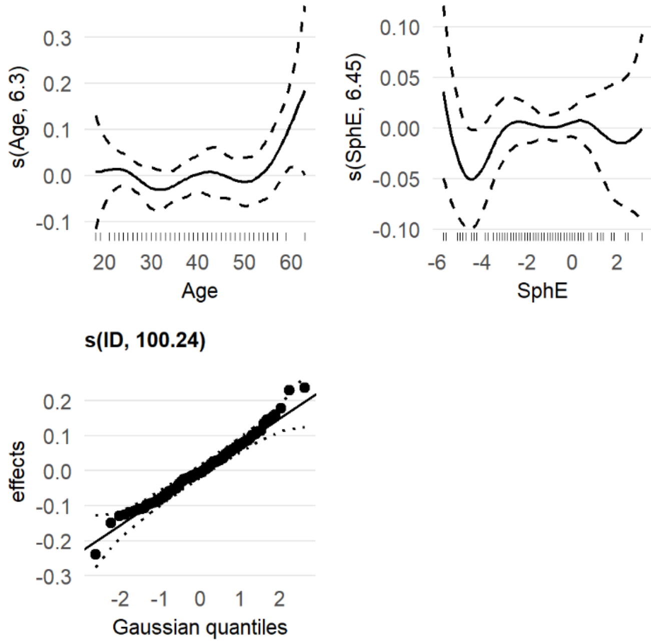

Dependent: FAZ size [mm^2]

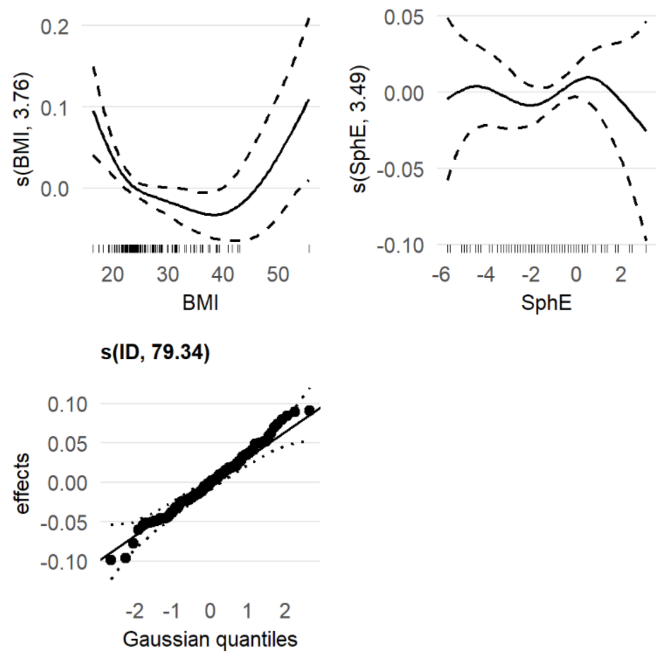

Dependent: Perfusion Density [mm^2/mm^2], Macula, Central Subfield

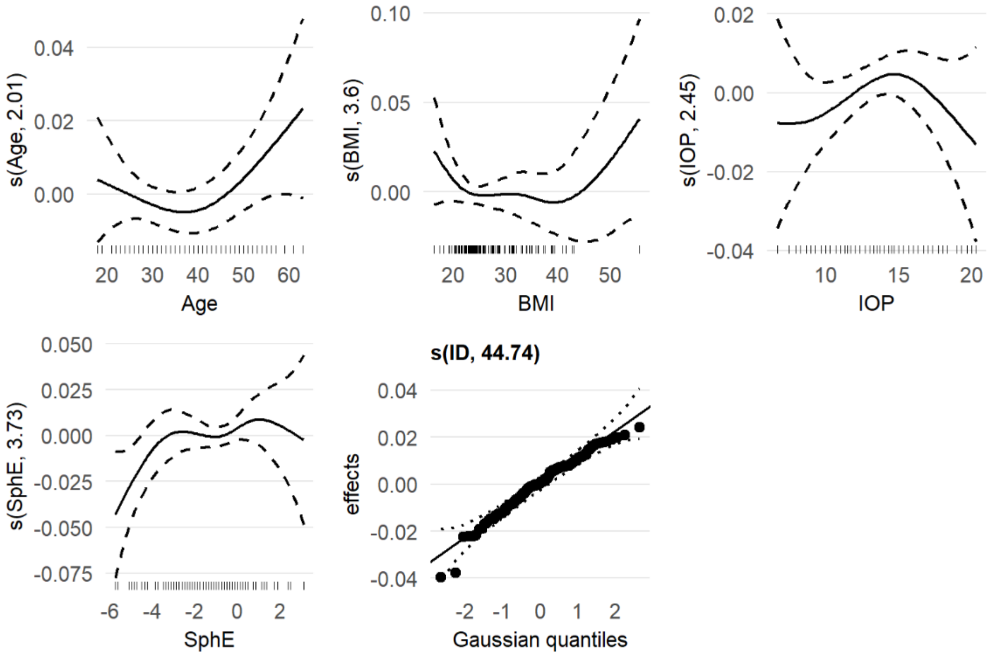

Dependent: Perfusion Density [mm^2/mm^2], Macula, Parafoveal Ring

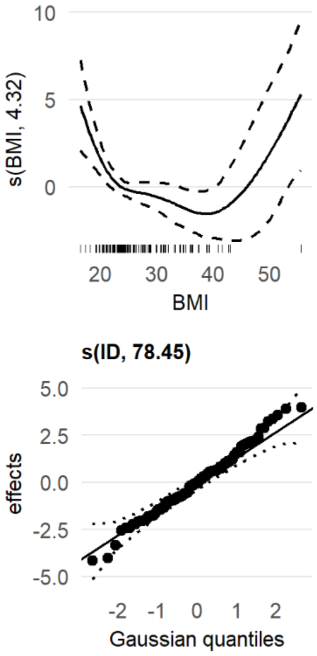

Dependent: Vessel Density [mm/mm^2], Macula, Central Subfield

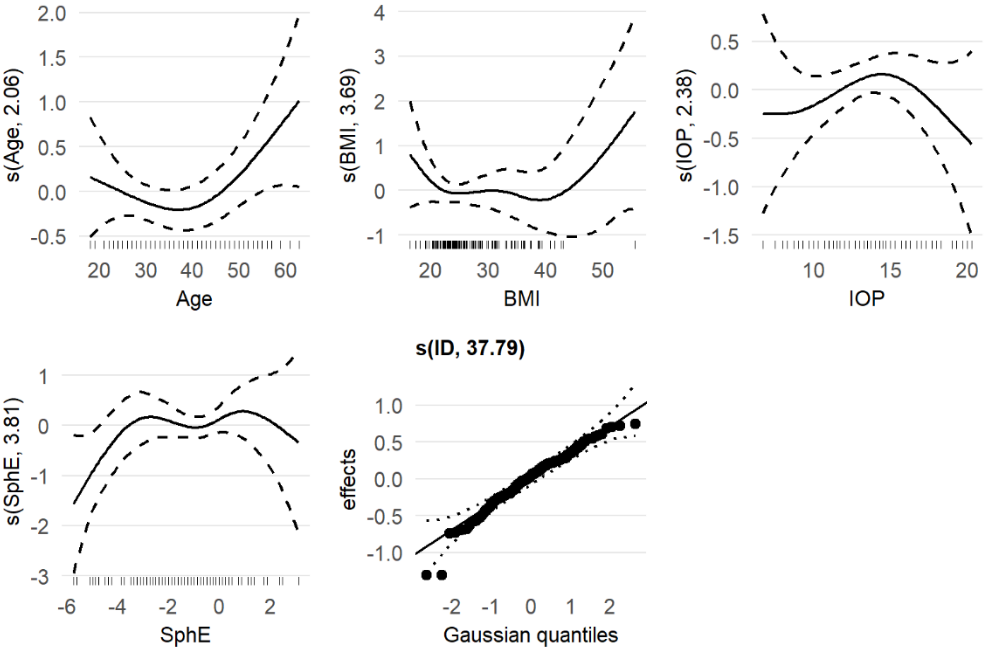

Dependent: Vessel Density [mm/mm^2], Macula, Parafoveal Ring

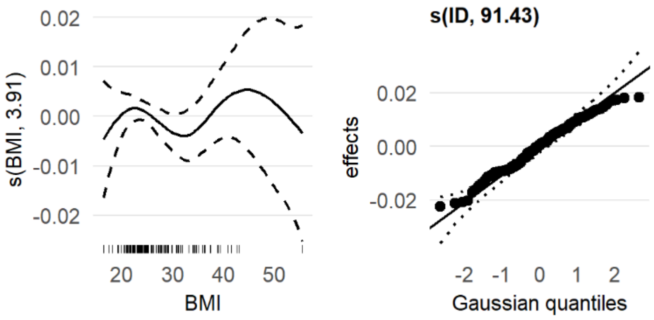

Dependent: Perfusion Density [mm^2/mm^2], Peripapillary
